# Supplementary material for: ’Well, it literally stops me from having a life when it’s really bad': a nested qualitative interview study of patient views on the use of self-management treatments for the management of recurrent sinusitis (SNIFS trial)
Source: BMJ Open. 2017 Nov 3;7(11):e017130. doi: 10.1136/bmjopen-2017-017130 (PMC5695339; doi:10.1136/bmjopen-2017-017130)
Supplement: Supplementary file 1 [file bmjopen-2017-017130supp001.pdf]

Appendix 1 – interview guide

SNIFS STUDY

Steam inhalation and Nasal Irrigation For recurrent Sinusitis

REC 07/Q1704/69

Part one: Experiences of Sinusitis

1. **Could you tell me how severe you feel your sinus problem is to you?**

   Prompts:
   A: What particularly bothers you about your sinus problems?
   B: Can you explain to me why this affects you?

2. **Could you tell me how often you become affected by your sinus problem?**

   Prompts:
   A: Are they a problem everyday?
   B: When did they first become a problem?
   C: Do you have times when your sinus symptoms don't affect you?
   D: Does your sinus problem prevent you from doing anything? (details of this/how does this make you feel).

3. **Could you tell me how you feel about visiting the doctors about your sinus problems?**

   Prompts:
   A: Have you seen any other healthcare professional about your symptoms? (nurse etc)
   B: Do you find it helpful to visit a doctor about your symptoms?

4. **Do you have any ideas about what could be causing your particular sinus problems?**

   Prompts
   A: Do you think it is something to do with how you are made?
   B: Do you think it is to do with bacteria and viruses?

Part two: Experiences and views of treatments

6. **What treatments have you used to treat your sinus problems in the past?**

   Prompts:
   A: Can you think of something that worked well?
   B: Can you think of something that didn't help at all?
   C: Where did you get the information from for these treatments?

7. **Before this study, had you ever heard about using nasal irrigation?**

   Prompts
   A: How do you feel about it?
   B: Do you think it would help to relieve symptoms?
   C: Do you think it might have any side effects?
   D: How often do you think it should be used for?
   E: How have you found using nasal irrigation?

8. **Had you heard about using steam inhalation to treat sinusitis before taking part in the study?**

   Prompts
   A: How do you feel about it?
   B: Do you think it would help to relieve symptoms?
   C: Do you think it might have any side effects?
   D: How often do you think it should be used for?
   E. How have you found using steam?

9. **Have you ever had antibiotics for your sinus problems?**

   Prompts
   A: How did you find using antibiotics?
   B: Would you consider using antibiotics again for your sinus problems?
   C: Have you heard of any problems with using antibiotics, such as side effects.
   D: Have you heard about bacteria becoming resistant to antibiotics?
   E: Where did you hear about these issues (tv, newspapers, GP, others etc)

   Conclusion

10. **Are there any other relevant issues we haven't covered that you would like to mention?**

11. **Are there any questions you that would like to ask me?**
