# Supplementary material for: ’Well, it literally stops me from having a life when it’s really bad': a nested qualitative interview study of patient views on the use of self-management treatments for the management of recurrent sinusitis (SNIFS trial)
Source: BMJ Open. 2017 Nov 3;7(11):e017130. doi: 10.1136/bmjopen-2017-017130 (PMC5695339; doi:10.1136/bmjopen-2017-017130)
Supplement: Author's manuscript [file bmjopen-2017-017130.draft_revisions.pdf]

BMJ Open is committed to open peer review. As part of this commitment we make the peer review history of every article we publish publicly available.

When an article is published we post the peer reviewers' comments and the authors' responses online. We also post the versions of the paper that were used during peer review. These are the versions that the peer review comments apply to.

The versions of the paper that follow are the versions that were submitted during the peer review process. They are not the versions of record or the final published versions. They should not be cited or distributed as the published version of this manuscript.

BMJ Open is an open access journal and the full, final, typeset and author-corrected version of record of the manuscript is available on our site with no access controls, subscription charges or pay-per-view fees (http://bmjopen.bmj.com).

If you have any questions on BMJ Open's open peer review process please email editorial.bmjopen@bmj.com

BMJ Open

'Well, it literally stops me from having a life when it's really bad ': a nested qualitative interview study of the decision to consult a GP or use self-management treatments for the management of recurrent sinusitis (SNIFS trial)

Journal:	*BMJ Open*
Manuscript ID	bmjopen-2017-017130
Article Type:	Research
Date Submitted by the Author:	03-Apr-2017
Complete List of Authors:	Leydon, Gerry; University of Southampton, Primary Care and Population Sciences<br>McDermott, Lisa; King's College London, Primary Care and Public Health Sciences<br>Thomas, Tammy; University of Southampton, PCPS Unit<br>Halls, Amy; University of Southampton, Primary Care and Population Sciences<br>Holdstock-Brown, Ben; University of Southampton, Primary Care and Population Sciences<br>Petley, Stephen; University of Southampton, Primary Care and Population Sciences<br>Wiseman, Clare; University of Southampton, Primary Care and Population Sciences<br>Little, Paul; University of Southampton, Primary Care and Population Science;
**Primary Subject Heading**:	General practice / Family practice
Secondary Subject Heading:	Qualitative research
Keywords:	PRIMARY CARE, QUALITATIVE RESEARCH, sinusitis, antibiotics, self help

SCHOLARONE™
Manuscripts

'Well, it literally stops me from having a life when it's really bad': a nested qualitative interview study of the decision to consult a GP or use self-management treatments for the management of recurrent sinusitis (SNIFS trial)

GM Leydon*[±], L McDermott**[±], T Thomas*, A Halls*, B Holdstock-Brown*, S Petley*, C Wiseman*, P Little*, on behalf of the SNIFS Investigators

[±]These authors to be regarded as Joint First Authors.

*Primary Care and Population Sciences, Faculty of Medicine, University of Southampton Aldermoor Health Centre, Aldermoor Close, Southampton, SO16 5ST

**Primary Care and Public Health Sciences, King's College, London 614, 6th Floor, Capital House, 42 Weston Street, London, SE1 3QD

Corresponding author:

Dr GM Leydon

gerry@soton.ac.uk

SNIFS Investigators:

University of Southampton: Paul Little, Tammy Thomas, Pat Alexant, Sophie Johnson, Lisa McDermott, Geraldine M. Leydon, Stephen Petley, Christ Smith, Clare Wiseman.

For peer review only

Abstract

Objective: To explore the experience and perceptions of illness, the decision to consult a GP and the use of self-management approaches for chronic or recurrent sinusitis.

Design: Qualitative semi-structured interview study.

Setting: UK Primary care.

Participants: 32 participants who had been participating in the 'SNIFS' (Steam-inhalation-and- Nasal-Irrigation-For-recurrent-Sinusitis) trial in the South of England.

Method: Thematic analysis of semi-structured, telephone interviews.

Results: Participants often reported dramatic impact on both activities and their quality of life. Participants were aware of both antibiotic side effects and resistance, but if they had previously been prescribed antibiotics, patients commonly believed that they would be necessary for the future treatment of sinusitis. Participants used self-help treatments for short and limited periods of time only, and did feel that regular and consistent use was necessary. Steam inhalation was described as acceptable, but commonly perceived as only beneficial in providing short-term relief, and by some participants only for severe symptoms. Nasal irrigation was viewed as acceptable and beneficial by more patients. However, some participants reported that they would not use the treatment again due to the uncomfortable side-effects they experienced, which outweighed any symptom relief which may have resulted had they continued.

Conclusions: Steam inhalation is acceptable but seen as having limited effectiveness. Nasal irrigation is generally acceptable and beneficial for symptoms, but detailed information on the correct procedure and potential benefits of persisting may increase acceptability and adherence in those patients who find it uncomfortable.

Strengths and limitations

1. The interviews permitted an exploration of participants' perceptions of recurrent or chronic sinusitis in a primary care setting, which is novel.
2. Trial participation may have led to a sample of participants particularly interested in this research, and therefore may not be representative of 'typical' patients.
3. Despite this, participants represented a sample of patients with recurrent sinusitis who had managed their condition using different treatment options.

For peer review only - http://bmjopen.bmj.com/site/about/guidelines.xhtml

Background

Respiratory tract infections (RTIs) are relatively brief and self-limiting conditions, however the prescribing of antibiotics for RTI's account for over 60% of all prescriptions in primary care[1] and around 54% of all consultations for RTIs[2]. Despite the large number of antibiotics frequently prescribed for RTIs, evidence has consistently demonstrated the limited benefit of antibiotics in treating these conditions[1,3,4]. Furthermore, the overuse of antibiotics can contribute to the spread of resistant bacteria[5,6], a problem which is currently on the increase and has been identified by the World Health Organisation as a serious issue which must be addressed with urgency[7].

The NICE guidelines recommend that antibiotics should not be prescribed for RTIs in most instances, unless a patient meets a specific at-risk criteria[1]. However, a number of 'self-help' treatments can be recommended to assist in relieving symptoms related to RTIs[1]. In relation to sinusitis (which is classified as a RTI), there is some evidence to show that both steam inhalation (involving a patient inhaling steam over a bowl of boiling water) and nasal irrigation (the irrigation of nasal cavities with saline) can reduce symptoms. However, the evidence is inconsistent and limited[8,9,10].

Previous qualitative work among patients with chronic sinusitis in secondary care suggests significant impact on quality of life and dissatisfaction with treatments received[11]. However, most recurrent or chronic sinusitis is managed in primary care, and we are aware of no qualitative studies to explore patients' perceptions in primary care. The SNIFS (Steam inhalation and Nasal Irrigation For recurrent Sinusitis) trial aimed to assess the efficacy of steam inhalation and nasal irrigation for the treatment of sinusitis. The study recruited 871 patients from across 72 practices in the South of England. The main findings from the trial have been published and demonstrate modest benefit at three and six months from nasal irrigation but not for steam inhalation[12]. This paper reports on the findings of a nested qualitative study which aimed to explore the feasibility and acceptability of these treatments from the patient perspective, the experience of illness and previous treatments, and factors which influence patient decisions to consult a GP or use self-help treatments (including steam inhalation and nasal irrigation) for the symptoms of sinusitis.

Methods

For peer review only - http://bmjopen.bmj.com/site/about/guidelines.xhtml

Participants and procedure

Participants were recruited from a sample of patients with chronic or recurrent sinusitis who were taking part in the SNIFS trial. Patients had seen the GP for previous episodes of sinusitis, and were also experiencing symptoms currently. Participants in the trial consented to being invited by telephone for an interview. The participants were recruited from areas across Southampton and Hampshire. Interview participants were purposively sampled to include a range of participants according to their allocation in the trial (e.g. steam inhalation/ nasal irrigation), and age and gender.

Interviews

Trained interviewers (LM, SP, BH, CS) conducted telephone interviews (in order to include a wide geographical area), with each lasting approximately half an hour. All interviews were audio–recorded and transcribed verbatim in preparation for analysis. Qualitative interviews provided the best method for gathering insights into participants' individual views about and experiences of treatments for sinusitis. A semi-structured interview guide included key topic areas while also providing flexibility to explore unanticipated issues.

Analysis

Inductive thematic analysis[13] was conducted on all transcripts to determine factors that influence patients' decision to consult a GP or use an alternative treatment for sinusitis. Following immersion in the transcripts, familiarisation was achieved and recurrent patterns and prominent themes were identified and labelled with codes. Each code label referred directly to the operationalisation of the theme content. A label and full descriptive definition was then provided for each theme. The codes and definitions were refined during a continuing process, which involved themes being linked, grouped, moved, re-labelled, added and removed to produce a set of themes and subthemes and a coding manual, which adequately fitted and thoroughly explained the data. The coding was iteratively developed across lead authors (led by LM and GL) and adjustments made where appropriate based on team discussion.

For peer review only - http://bmjopen.bmj.com/site/about/guidelines.xhtml

Findings

Participants:

In total 32 participants took part in the study. The age of participants ranged from 18 to 74 (mean age 55). Approximately 72% (23) were women and 28% (9) men.

Themes:

Thematic analysis identified a total of six themes relating to using self-help treatments (steam inhalation/nasal irrigation/other remedies) or consulting with a GP for the treatment of sinusitis. Illustrative quotations are provided and details relating to the interviewee are provided in parentheses.

Table 1: Themes identified in analysis

Themes	Sub-categories
**1. Perceptions of severity**	*-Duration of symptoms*<br>*-Perceived signs of severity*<br>*-Quality of life impact*
**2. Advice from others**	*-Acceptance of GP advice*<br>*-Consideration of alternative advice*
**3. Perceptions of antibiotics**	*-Antibiotics have unpleasant side-effects*<br>*-Concerns about resistance*<br>*-Previous experience of antibiotics.*
**4. Perceptions of self-help treatment**	*-Previous experience of treatments*<br>*-Treatment duration short and irregular*
**5. Experiences of steam inhalation**	*-Beneficial but only for short term relief*<br>*-Beneficial but only for severe symptoms*
**6. Experiences of nasal irrigation**	*-Strong benefits of treatment outweigh any discomfort*<br>*-Uncomfortable side-effects of treatment cannot justify use*

1. Perceptions of severity

Perceptions of severity were shaped by the duration of symptoms, perceived indicators of severity, and particularly by the impact of the illness on quality of life.

For peer review only - http://bmjopen.bmj.com/site/about/guidelines.xhtml

Duration of symptoms

Most participants reported that an evaluation of severity was based on the number of days that they had experienced symptoms, which they related to the current attack (this ranged from a few days to weeks).

> *"It was horrible, **I tried to cope for about a week on my own** but in the end **I just had to go** (to the GP)" (Partipant L05).*

Perceived signs of severity

A number of factors were used as indicators of severity including a variety of signs and symptoms ranging from pain (head, sinuses, face) to feelings (e.g. nasal passages being blocked), noises (from nose) and sensations (around face and head).

> *"It's just the….**like my face hurting, my headaches and just the…..lethargic** and you know just everything about it really" (Participant L04).*

Quality of life impact

Participants discussed the way in which their symptoms had prevented them from attending work, caring for their children, and taking part in social or other activities.

> *"Well, I don't tend to go swimming or anything like that, or a lot of exercise. And even gardening, things where I have to put my head forward, you know, my brain feels like it's bouncing inside my head. So I don't tend to do that sort of thing" (Participant B03).*

Others described the sinusitis as having an impact on life in general.

> *"Well, it literally stops me from having a life when it's really bad because I really can't get up and walk about. So it does interfere with my life" (Participant B10).*

2. Advice from others

For peer review only - http://bmjopen.bmj.com/site/about/guidelines.xhtml

Acceptance of GP advice

Overwhelmingly, most participants reported that they were happy to accept and follow their GP's treatment advice. This advice appeared to be accepted regardless of previous experiences or perceptions which may be held regarding their condition or appropriate treatment options (such as the need for antibiotics). This advice could include taking antibiotics or using various self-help treatments such as nasal irrigation or steam inhalation.

> *"If I was advised to [use steam inhalation], yes. I mean, it's probably not something I would think about just getting on and doing, but if I was asked- **if a GP suggested it then, yes I would**" (Participant L06).*

Consideration of alternative advice

Sources other than their GP were commonly used in decision making, predominantly family members, but also the internet and newspapers.

> *"We had **relatives** over from Australia **who brought me a bottle** (of eucalyptus oil to inhale) and that's fantastic, that's for a general cold as well" (Participant L05).*

3. Perceptions of antibiotics

Individuals' perceptions of antibiotics were related to beliefs about side effects, concerns about resistance, and their previous experience (good or bad) of taking antibiotics.

Antibiotics have unpleasant side-effects

Many participants were well aware of side effects, mainly related to stomach complaints, although could include a variety of effects such as skin rashes, or mouth ulcers.

> "*It can give you an upset stomach, well I've experienced it myself, because doesn't it destroy the good bacteria or something?" (Participant B08).*

> *"Well, ………..they (antibiotics) upset my stomach sometimes." (Participant B10).*

For peer review only - http://bmjopen.bmj.com/site/about/guidelines.xhtml

"I think some people I've met can't take them, they come out in ***quite a severe rash*** *sometimes, and I have noticed, perhaps like tiny* ***ulcers appearing in my mouth****, which I've found does happen with antibiotics sometimes" (Participant L02)*

Concerns about resistance

Many participants were also aware of, and worried about, the issue of antibiotic resistance, but commonly described it as the body becoming resistant.

"We're ***building up a resistance*** *to them and everything. So I do know there is a problem using them. I felt quite worried when I had to take 2, 100 milligrams in a week" (Participant B10).*

"I know it's not the answer because we're all ***getting immune*** *to them... I am very aware that we can get, well, you know, resistant to antibiotics" (Participant B05).*

Previous experience of antibiotics

Patients who attributed symptom resolution to antibiotics believed they would be effective and to want to take them again for the same symptoms.

"I've had a course (of antibiotics) from the doctor *and they've been ineffective, and gone back and given me a stronger dose and* ***it does help****" (Participant L03).*

"Well, only the antibiotics, was the only thing that got rid of it completely" (Participant B04).

"Very often the only thing I can have is an antibiotic to cure it" (Participant B05).

4. Perceptions of self-help treatment

Previous experience of treatments

Previous experience of using self-help treatments also strongly influenced their decision of whether or not to use them again, consult a GP, or try other self-help methods.

For peer review only - http://bmjopen.bmj.com/site/about/guidelines.xhtml

*“**Done it (steam inhalation) all my life over the years, so no, no, not a problem doing things like that.** It’s the way- to be buried under a towel…” (Participant L07).*

“It’s (inhalation) something I’ve done for quite a few years with them” (Participant B03).

Treatment duration short and irregular

Some participants reported that in general they used self-help treatments for short and limited periods of time only, judging regular and consistent use as unnecessary. Self-help treatments appeared to be used in an irregular and inconsistent way (e.g. stopping treatment (such as steam inhalation) as soon as relief is first experienced.

*Well I’ve **never used it** (steam inhalation) **more than probably half a dozen times** for one session (illness) over a period of days” (Participant L09)*

5. Experiences of steam inhalation

Beneficial but only for short-term relief

Most participants who had experienced steam inhalation - either as part of the trial, following previous advice from a GP, or as an independent technique which the participant had previously tried - reported that the treatment could be beneficial in relieving symptoms. However, accounts signalled a belief that symptoms were only reduced for a short period of time (up to a few hours).

*“That steam inhalation does actually provide immediate- **it clears the airways, but, again, it just doesn’t last.** Even if I do it on a regular basis, it’s like- you know, **within a very short period after doing the inhalation I’m blocked up again**…within half an hour” (Participant L05).*

Beneficial but only for severe symptoms

Paradoxically some participants who had experienced steam inhalation reported the belief that steam was only beneficial in relieving the most severe symptoms of congestion caused by sinusitis and that it had little effect if symptoms were only mild.

For peer review only - http://bmjopen.bmj.com/site/about/guidelines.xhtml

"I haven't used it (steam inhalation) this time, I suppose ***it's (sinusitis) not bad enough to use*** *that,* ***but if I couldn't sleep and that, then I would*** *have to do that (inhale steam)" (Participant L04).*

*"****I only do it (steam inhalation) if I'm feeling really bad****, if it's getting really bad" (Participant L08)*

6. Experiences of nasal irrigation

Participants' experiences of using nasal irrigation also influenced their decisions to use the method again or whether to consult a GP. In similar fashion to steam inhalation the treatment of nasal irrigation had been experienced by participants either as part of the trial, but for some following previous advice from a GP, or as an independent technique that the participant had tried previously. Unsurprisingly it was clear that the balance of discomfort and symptomatic benefit influenced their use of irrigation.

Strong benefits of treatment outweigh any discomfort

Approximately half of the participants who had experienced nasal irrigation reported that the technique provided relief for their symptoms and that although the treatment may be slightly uncomfortable at times, the benefits received outweighed any discomfort suffered.

"I can put up with it because it does improve the sinus condition" (Participant C01).

"After the initial shock of having to do it, it does help. It helped relieve symptoms…It freaked me out a bit to start with. But, once you get into the hang of it, it's alright" (Participant B09).

Uncomfortable side-effects of treatment cannot justify use

However, around half of the participants who experienced nasal irrigation reported that the treatment was too uncomfortable or unpleasant. Side-effects reported included; shuddering; an increase in the feeling of mucus being produced, water in the back of the throat, water running out of the nose hours later, and general pain.

*"One of my memories is of the actual shuddering and in the end **I just gave up because I found it very unpleasant to do**" (Participant L01).*

"It doesn't actually drain it, you can actually blow your nose and get rid of it. It goes everywhere. It goes down the back of your throat and you end up coughing" (Participant B01).

"I don't know whether it's the configuration of the sinuses or whatever, but instead of, sort of, coming out the other side, it was going straight down the tubes into my ears. And, well, that was quite uncomfortable" (Participant B03).

Discussion

We are aware of no prior qualitative studies of patients' perceptions of recurrent or chronic sinusitis in a primary care setting, nor of perceptions of steam inhalation and nasal irrigation. The study identified six key themes related to factors that influence patients' decisions to consult a GP or use a self-help treatment (in particular steam inhalation or nasal irrigation) for symptoms of sinusitis. Findings are discussed in relation to the most prevalent and influential themes outlined by participants.

Main findings

Patients with recurrent or chronic sinusitis described the often dramatic impact on their activities and quality of life, and viewed their sinusitis as a chronic condition. They based most treatment decisions on past experiences of managing symptoms. Thus, although participants were well aware of the limitations of antibiotics and did not expect to receive them every time they consulted a GP, if they had previously been prescribed antibiotics, they often believed they would be necessary for the future treatment of sinusitis. Patients used self-help treatments for short and limited periods of time only, and did not feel that regular and consistent use was necessary. Steam inhalation was viewed as an acceptable treatment option, however this was commonly perceived as a method which was only beneficial in providing short term relief, and paradoxically some participants also believed it was only helpful for the most severe symptoms. Many participants viewed nasal irrigation as acceptable and beneficial, but some would not use the treatment again due to the balance of uncomfortable or unpleasant side-effects which outweighed any symptom relief.

For peer review only - http://bmjopen.bmj.com/site/about/guidelines.xhtml

Comparison with existing literature

If participants had been prescribed antibiotics in the past for the treatment of sinusitis, they attributed symptom benefit to antibiotics and accounts indicated belief that this treatment would, therefore, be the optimal approach for future management of the condition. In addition, participants also reported a strong acceptance of any GP advice, also suggesting that if GPs prescribe antibiotics for sinusitis, patients' beliefs that antibiotics may be needed in the future will be strongly reinforced. These findings are supported by previous quantitative research which has documented that prescribing antibiotics for RTIs directly influences patients' views relating to the need to consult a GP and take antibiotics for future RTIs[14]. The 'medicalisation' of illness due to the overprescribing of antibiotics for RTIs such as sinusitis helps to reinforce high prescribing of antibiotics despite evidence of their limited symptom benefit and NICE recommendations[1,3,4].

However, the medicalisation of illness cannot easily explain the fact that the rates of prescribing for sinusitis are the highest of any respiratory infection – with more than 90% of individuals still receiving antibiotics[2]. One factor maintaining high prescription rates is likely to be the significant impact of the condition on the patient's quality of life – described in this study in such dramatic terms as 'horrible' and 'it literally stops me from having a life'. The significance of impact of quality of life is supported by similar findings from a secondary care sample[11]. Another is that sinusitis is second only to chest infections in a long duration of each attack – on average just short of three weeks[15]. Thus, given an unpleasant and long lasting condition, if GPs have nothing else to recommend antibiotics are likely to be used.

However, patients were aware of the limitations of antibiotics and would be happy to accept GP advice not to take them, if this was recommended. This lack of expectation for antibiotics has also been reported in previous research, and in particular often contrasts with GP perceptions of high levels of patient pressure to prescribe (e.g. 14,16). Therefore, the findings suggest that if GPs could reduce their prescribing of antibiotics for sinusitis, many patients will be happy to accept this and may be less likely to believe that a GP consultation or antibiotics would be necessary for managing their sinusitis in the future.

Implications of using steam inhalation and nasal irrigation in clinical practice

For peer review only - http://bmjopen.bmj.com/site/about/guidelines.xhtml

Overall, patients reported that steam inhalation was an acceptable treatment for the symptoms of sinusitis. However, many patients believed that steam inhalation was only beneficial for the short-term relief of symptoms and this corresponds with findings from the main clinical trial which found limited benefit[12]. Whilst clearly some patients will feel they get benefit from steam inhalation there is also some evidence form other studies of mild thermal injury using steam[17]. Overall, the combined qualitative and quantitative findings suggest that there is a limited place for routinely advising patients to use steam inhalation for chronic or recurrent sinusitis.

Patients who had experienced nasal irrigation could be identified in one of two sub-groups: those who described irrigation as an acceptable technique which could relieve their symptoms; and those who described the discomfort experienced during irrigation with limited justification for its use as a treatment. Patients who reportedly found irrigation to be acceptable often reported that they had persisted with it despite initially finding the treatment very uncomfortable, and many reported how they had 'got used to' the discomfort in order to experience the benefits. Therefore, it is possible that the patients who found irrigation unacceptable may have changed their mind if they had persisted with the treatment. In addition, patients who reported irrigation as being unacceptable tended to report side-effects such as water going down their throat. This suggests that some of these patients may not have been using the correct technique when conducting the procedure and with further guidance and instruction may have been able to continue. In short, findings suggest that nasal irrigation can be viewed as an acceptable treatment for the symptoms of sinusitis. An important caveat to this, however, is the need for detailed and clear patient information on the correct procedure and the potential benefits of persisting with the technique in terms of finding it easier with increased practice/use of nasal irrigation. This supports findings from the main trial that demonstrated modest benefit at 3 and 6 months from nasal irrigation.

Strengths and limitations

One of the key strengths of the study lies in the fact that all patients interviewed had experienced at least one of the treatment options (steam inhalation or nasal irrigation) as part of the SNIFS trial. Therefore, the interviewees could discuss in-depth the various treatment options in accordance with the way in which the treatments were delivered during the trial. This strengthens the findings and allows stronger conclusions to be drawn.

For peer review only - http://bmjopen.bmj.com/site/about/guidelines.xhtml

However, the sample of participants who took part in the interviews may have limited the scope of the findings due to the fact that they had all consented to and taken part in the SNIFS trial. Trial participation may have led to a sample of participants who were particularly interested in research of this nature and may not have represented views held by 'typical' or non-trial patients. In particular, participants may have held more positive views towards self-management treatments due to their interest and participation in research of this nature.

Despite this possible 'research biased' sample, all patients interviewed did represent a sample of patients who had experienced recurrent sinusitis and had also managed their condition using a number of treatment options over the years. In addition, a few patients interviewed had also withdrawn from the trial but were happy to take part in an interview; suggesting that a wider range of patient views were included within the sample (and not simply those who may view self-management treatments in a more positive light).

Conclusion

The findings suggest that steam inhalation is viewed as an acceptable treatment which patients are happy to use, but with limited short-term benefit. Nasal irrigation is acceptable to many patients in relieving symptoms, but some find it uncomfortable or mildly unpleasant. However, detailed information on the correct procedure and potential benefits of persisting with the technique may increase the acceptability of nasal irrigation in patients who find it initially uncomfortable.

Contributorship

GM Leydon led the qualitative work as part of the SNIFS trial. P Little secured research funding/and acted as overall PI of the SNIFS trial. T Thomas facilitated coordination and recruitment with P Alexant and S Johnson. S Petley, B Holdstock-Brown, C Smith and C Wiseman collected interview data. All authors were involved in/commented on data analysis (led by G Leydon and L McDermott). AH progressed the manuscript and all authors contributed to its writing.

For peer review only - http://bmjopen.bmj.com/site/about/guidelines.xhtml

Funding

The study was funded by a grant from the National Institute for Health Research (NIHR) under its Programme Grants for Applied Research (grant no. RP-PG-0407-10098). The views expressed are those of the authors and not necessarily those of the National Health Service, the NIHR or the Department of Health. The University of Southampton was the sponsor, but it had no role in the running of the study, the analysis or interpretation of the results, or the preparation of the manuscript.

Competing interests statement

There are no competing interests.

Acknowledgements

The authors are grateful to the patients and health care professionals who contributed their time and effort and helpful insights to make the study possible. They are also grateful to the Programme Steering Committee for their support and advice throughout the study; NIHR for funding Geraldine Leydon on a personal Fellowship during this study; and C Smith who contributed to the research whilst a medical student.

Data sharing statement

There are no data sets available for sharing.

References

1. Tan T, Little P, Stokes T: Antibiotic prescribing for self limiting respiratory tract infections in primary care: summary of NICE guidance. *BMJ.* 2008, *337:*a437
2. Gulliford MC, Dregan A, Moore MV, et al.: Continued high rates of antibiotic prescribing to adults with respiratory tract infection: survey of 568 UK general practices. *BMJ Open.* 2014;*4:*e006245.
3. Little P, Del Mar C, Gould C, et al.: Primary carePredictors of poor outcome and benefits from antibiotics in children with acute otitis media: pragmatic randomised trialCommentary: research directions for treatment for acute otitis media. *BMJ.* 2002;*325:*22.

For peer review only - http://bmjopen.bmj.com/site/about/guidelines.xhtml

4. Macfarlane J, Holmes W, Gard P, et al.: Prospective study of the incidence, aetiology and outcome of adult lower respiratory tract illness in the community. *Thorax.* 2001;*56:*109-114.
5. Dagan R, Leibovitz E, Greenberg D, et al.: Dynamics of pneumococcal nasopharyngeal colonization during the first days of antibiotic treatment in pediatric patients. *Pediatr Infect Dis J.* 1998;*17:*880-885.
6. Costelloe C, Metcalfe C, Lovering A, Mant D, Hay AD: Effect of antibiotic prescribing in primary care on antimicrobial resistance in individual patients: systematic review and meta-analysis. *BMJ.* 2010;*340:*c2096.
7. Leung E, Weil DE, Raviglione M, Nakatani H: The WHO policy package to combat antimicrobial resistance. *Bulletin of the World Health Organization.* 2011, *89:*390-392.
8. Kassel JC, King D, Spurling GK: Saline nasal irrigation for acute upper respiratory tract infections. *Cochrane Database Syst* Rev. 2010; 17(3):CD006821
9. Tomooka LT, Murphy C, Davidson TM: Clinical study and literature review of nasal irrigation. *Laryngoscope.* 2000;*110:*1189-1193.
10. Benninger MS, Anon J, Mabry RL: The medical management of rhinosinusitis. *Otolaryngol Head Neck Surg.* 1997, *117:*S41-S49.
11. Erskine S, Verkerk M, Notley C, Williamson I, Philpott C: Chronic rhinosinusitis: patient experiences of primary and secondary care–a qualitative study. *Clinical Otolaryngology.* 2016, *41:*8-14.
12. Little P, Stuart B, Mullee M, et al.: Effectiveness of steam inhalation and nasal irrigation for chronic or recurrent sinus symptoms in primary care: a pragmatic randomized controlled trial. *Can. Med. Assoc. J.* 2016;*188:*940-949.
13. Braun V, Clarke V: Using thematic analysis in psychology. *Qual Red Psychol* 2006;*3:*77-101.
14. Little P, Rumsby K, Kelly J, et al.: Information leaflet and antibiotic prescribing strategies for acute lower respiratory tract infection: a randomized controlled trial. *JAMA.* 2005, *293:*3029-3035.
15. Thompson M, Vodicka TA, Blair PS, et al.: Duration of symptoms of respiratory tract infections in children: systematic review. *BMJ.* 2013, *347:*f7027.
16. Leydon GM, McDermott L, Moore M, et al.: A qualitative study of GP, NP and patient views about the use of rapid streptococcal antigen detection tests (RADTs) in primary care:‘swamped with sore throats?’. *BMJ Open.* 2013, *3:*e002460.
17. Little P, Moore M, Kelly J, et al.: Ibuprofen, paracetamol, and steam for patients with respiratory tract infections in primary care: pragmatic randomised factorial trial. *BMI* 2013;347:f6041.

For peer review only

COREQ (COnsolidated criteria for REporting Qualitative research) Checklist

A checklist of items that should be included in reports of qualitative research. You must report the page number in your manuscript where you consider each of the items listed in this checklist. If you have not included this information, either revise your manuscript accordingly before submitting or note N/A.

Topic	Item No.	Guide Questions/Description	Reported on Page No.
**Domain 1: Research team and reflexivity**			
Personal characteristics			
Interviewer/facilitator	1	Which author/s conducted the interview or focus group?	4
Credentials	2	What were the researcher's credentials? E.g. PhD, MD	
Occupation	3	What was their occupation at the time of the study?	
Gender	4	Was the researcher male or female?	
Experience and training	5	What experience or training did the researcher have?	
Relationship with participants			
Relationship established	6	Was a relationship established prior to study commencement?	
Participant knowledge of the interviewer	7	What did the participants know about the researcher? e.g. personal goals, reasons for doing the research	
Interviewer characteristics	8	What characteristics were reported about the inter viewer/facilitator? e.g. Bias, assumptions, reasons and interests in the research topic	
**Domain 2: Study design**			
Theoretical framework			
Methodological orientation and Theory	9	What methodological orientation was stated to underpin the study? e.g. grounded theory, discourse analysis, ethnography, phenomenology, content analysis	
Participant selection			
Sampling	10	How were participants selected? e.g. purposive, convenience, consecutive, snowball	
Method of approach	11	How were participants approached? e.g. face-to-face, telephone, mail, email	4
Sample size	12	How many participants were in the study?	5
Non-participation	13	How many people refused to participate or dropped out? Reasons?	
Setting			
Setting of data collection	14	Where was the data collected? e.g. home, clinic, workplace	4
Presence of non-participants	15	Was anyone else present besides the participants and researchers?	
Description of sample	16	What are the important characteristics of the sample? e.g. demographic data, date	
Data collection			
Interview guide	17	Were questions, prompts, guides provided by the authors? Was it pilot tested?	
Repeat interviews	18	Were repeat inter views carried out? If yes, how many?	
Audio/visual recording	19	Did the research use audio or visual recording to collect the data?	4
Field notes	20	Were field notes made during and/or after the inter view or focus group?	
Duration	21	What was the duration of the inter views or focus group?	4
Data saturation	22	Was data saturation discussed?	
Transcripts returned	23	Were transcripts returned to participants for comment and/or	

For peer review only

Topic	Item No.	Guide Questions/Description	Reported on Page No.
		correction?	
**Domain 3: analysis and findings**			
Data analysis			
Number of data coders	24	How many data coders coded the data?	4
Description of the coding tree	25	Did authors provide a description of the coding tree?	
Derivation of themes	26	Were themes identified in advance or derived from the data?	5
Software	27	What software, if applicable, was used to manage the data?	
Participant checking	28	Did participants provide feedback on the findings?	
Reporting			
Quotations presented	29	Were participant quotations presented to illustrate the themes/findings? Was each quotation identified? e.g. participant number	6 onwards
Data and findings consistent	30	Was there consistency between the data presented and the findings?	6 onwards
Clarity of major themes	31	Were major themes clearly presented in the findings?	6 onwards
Clarity of minor themes	32	Is there a description of diverse cases or discussion of minor themes?	

Developed from: Tong A, Sainsbury P, Craig J. Consolidated criteria for reporting qualitative research (COREQ): a 32-item checklist for interviews and focus groups. *International Journal for Quality in Health Care*. 2007. Volume 19, Number 6: pp. 349 – 357

Once you have completed this checklist, please save a copy and upload it as part of your submission. DO NOT include this checklist as part of the main manuscript document. It must be uploaded as a separate file.

For peer review only

BMJ Open

'Well, it literally stops me from having a life when it's really bad ': a nested qualitative interview study of patient views on the use of self-management treatments for the management of recurrent sinusitis (SNIFS trial).

Journal:	*BMJ Open*
Manuscript ID	bmjopen-2017-017130.R1
Article Type:	Research
Date Submitted by the Author:	19-May-2017
Complete List of Authors:	Leydon, Gerry; University of Southampton, Primary Care and Population Sciences<br>McDermott, Lisa; King's College London, Primary Care and Public Health Sciences<br>Thomas, Tammy; University of Southampton, PCPS Unit<br>Halls, Amy; University of Southampton, Primary Care and Population Sciences<br>Holdstock-Brown, Ben; University of Southampton, Primary Care and Population Sciences<br>Petley, Stephen; University of Southampton, Primary Care and Population Sciences<br>Wiseman, Clare; University of Southampton, Primary Care and Population Sciences<br>Little, Paul; University of Southampton, Primary Care and Population Science;
**Primary Subject Heading**:	General practice / Family practice
Secondary Subject Heading:	Qualitative research
Keywords:	PRIMARY CARE, QUALITATIVE RESEARCH, sinusitis, antibiotics, self help

SCHOLARONE™
Manuscripts

'Well, it literally stops me from having a life when it's really bad': a nested qualitative interview study of patient views on the use of self-management treatments for the management of recurrent sinusitis (SNIFS trial)

GM Leydon*±, L McDermott**±, T Thomas*, A Halls*, B Holdstock-Brown*, S Petley*, C Wiseman*, P Little*, on behalf of the SNIFS Investigators

±These authors to be regarded as Joint First Authors.

*Primary Care and Population Sciences, Faculty of Medicine, University of Southampton
Aldermoor Health Centre, Aldermoor Close, Southampton, SO16 5ST

**Primary Care and Public Health Sciences, King's College, London
614, 6th Floor, Capital House, 42 Weston Street, London, SE1 3QD

Corresponding author:
Dr GM Leydon
gerry@soton.ac.uk

SNIFS Investigators:
University of Southampton: Paul Little, Tammy Thomas, Pat Alexant, Sophie Johnson, Lisa McDermott, Geraldine M. Leydon, Stephen Petley, Chris Smith, Clare Wiseman.

For peer review only - http://bmjopen.bmj.com/site/about/guidelines.xhtml

Abstract

Objective: To explore the experience and perceptions of illness, the decision to consult a GP and the use of self-management approaches for chronic or recurrent sinusitis.

Design: Qualitative semi-structured interview study.

Setting: UK Primary care.

Participants: 32 participants who had been participating in the 'SNIFS' (Steam-inhalation-and- Nasal-Irrigation-For-recurrent-Sinusitis) trial in the South of England.

Method: Thematic analysis of semi-structured, telephone interviews.

Results: Participants often reported dramatic impact on both activities and their quality of life. Participants were aware of both antibiotic side effects and resistance, but if they had previously been prescribed antibiotics, many patients believed that they would be necessary for the future treatment of sinusitis. Participants used self-help treatments for short and limited periods of time only, and did feel that regular and consistent use was necessary. In the context of the trial, steam inhalation used for recurrent sinusitis was described as acceptable, but is seen as having limited effectiveness. Nasal irrigation was viewed as acceptable and beneficial by more patients. However, some participants reported that they would not use the treatment again due to the uncomfortable side-effects they experienced, which outweighed any symptom relief which may have resulted had they continued.

Conclusions: Steam inhalation is acceptable but seen as having limited effectiveness. Nasal irrigation is generally acceptable and beneficial for symptoms, but detailed information on the correct procedure and potential benefits of persisting may increase acceptability and adherence in those patients who find it uncomfortable.

Strengths and limitations

1. The interviews permitted an exploration of participants' perceptions of particular management strategies for recurrent or chronic sinusitis.
2. Participants represented a sample of patients with recurrent sinusitis who had managed their condition using different treatment options.
3. Trial participation may have led to a sample of participants particularly interested in this research, and therefore may not be representative of 'typical' patients.

DRAFT For peer review only

4. Due to study procedure limitations the team was unable to collect characteristics of all participants and this has reduced our ability to analyse data according to key characteristics, such as trial arm, in comparative ways.

Background

Respiratory tract infections (RTIs) are self-limiting conditions, lasting around one to two weeks. However antibiotic prescriptions for RTIs account for over 60% of all antibiotic prescriptions in primary care[1]. One study of general practice prescribing found antibiotics to be prescribed in around 54% of all consultations for RTIs[2]. Despite the large number of antibiotics frequently prescribed for RTIs, evidence has consistently demonstrated the limited benefit of antibiotics in treating these conditions[1,3,4]. Furthermore, the overuse of antibiotics can contribute to the spread of resistant bacteria[5,6]. This problem is currently on the increase and has been identified by the World Health Organisation as a serious issue which must be addressed with urgency[7].

Whilst antibiotics may be beneficial for some patients with sinusitis, the NICE guidelines recommend that antibiotics should not be prescribed for RTIs in most instances, unless a patient meets a specific at-risk criteria, such as being systematically unwell, or at high risk of serious complications due to a pre-existing comorbidity[1]. However, a number of 'self-help' treatments can be recommended to assist in relieving symptoms related to RTIs[1]. In relation to sinusitis (which is classified as a RTI), there is some evidence to show that both steam inhalation (involving a patient inhaling steam over a bowl of boiling water) and nasal irrigation (the irrigation of nasal cavities with saline) can reduce symptoms. However, the evidence is inconsistent and limited[8,9,10].

Previous qualitative work among patients with chronic sinusitis in secondary care suggests significant impact on quality of life and dissatisfaction with treatments received[11]. However, most recurrent or chronic sinusitis is managed in primary care, and we are aware of no qualitative studies to explore patients' perceptions in primary care. The SNIFS (Steam inhalation and Nasal Irrigation For recurrent Sinusitis) trial aimed to assess the efficacy of steam inhalation and nasal irrigation for the treatment of sinusitis. The study recruited 871 patients from across 72 practices in the South of England. Participants were randomly assigned to an advice strategy: to receive advice to use nasal saline irrigation daily, or no such advice; to receive advice to use steam inhalation daily, or no such advice. A

For peer review only - http://bmjopen.bmj.com/site/about/guidelines.xhtml

combination group included participants randomly assigned to receive advice to perform daily nasal irrigation and daily steam inhalation. This advice is shown in Table 1. All study participants had access to usual care. Participants had had at least two episodes of acute sinusitis or one episode of chronic sinusitis (lasting for 12 weeks or more) in the three years before enrolment[12].

Table 1

Advice strategy	Definition
Nasal saline irrigation	Participants given verbal instructions and a link to a demonstration video on YouTube. Participants provided with a neti pot and asked to irrigate their nose daily for six months.
Steam inhalation	Participants were asked to inhale steam for five minutes each day.
Usual care	Use of medications or referral was at discretion of patient's physician.

The main findings from the trial have been published and demonstrate modest benefit at three and six months from nasal irrigation but not for steam inhalation[12]. This paper reports on the findings of a nested qualitative study which aimed to explore the feasibility and acceptability of these treatments from the patient perspective, the experience of illness and previous treatments, and factors which influence patient decisions to consult a GP or use self-help treatments (including steam inhalation and nasal irrigation) for the symptoms of sinusitis.

Methods

Participants and procedure

Participants were recruited from patients with chronic or recurrent sinusitis who were taking part in the SNIFS trial. Patients had seen the GP for previous episodes of sinusitis, and were also experiencing symptoms currently, although they did not have any 'at risk criteria' as this would have excluded them from participating in the trial. Participants in the trial consented to being invited by telephone for an interview. Participants who had withdrawn from the trial were also eligible to take part in the interviews. The participants were recruited from areas across Southampton and Hampshire. Interview participants were purposively sampled to include a range of participants according to their allocation in the trial (e.g. steam inhalation/ nasal irrigation), and age and gender.

For peer review only - http://bmjopen.bmj.com/site/about/guidelines.xhtml

Interviews

Trained interviewers LM and CW (female) and SP and BH (male) conducted telephone interviews (in order to include a wide geographical area), with each lasting approximately half an hour. Ethical approval was in place for interviews to last up to 60 minutes and no interviews exceeded this. SP, BH and CW were medical students during their involvement in this research, and supervised by senior academics (GL and PL). All interviews were audio–recorded and transcribed verbatim in preparation for analysis. Semi-structured qualitative interviews allowed researchers to gather insights into participants' individual views and experiences of treatments for sinusitis, as well as providing a structure for comparison across different accounts[13]. The interview guide was developed by the research team to ensure our aims were met. Previous literature was reviewed and questions thought to be relevant included. The guide was tested, refined and agreed. It included key topic areas while also providing flexibility to explore unanticipated issues. Subtle realism best characterises our epistemological position[14].

Analysis

Inductive thematic analysis[15] was conducted on all transcripts to determine factors that influence patients' decision to consult a GP or use an alternative treatment for sinusitis, as well as being open to themes outside of the core aims of the study. Following immersion in the transcripts, familiarisation was achieved and recurrent patterns and prominent themes were identified and labelled with codes. Each code label referred directly to the operationalisation of the theme content. A label and full descriptive definition was then provided for each theme. The codes and definitions were refined during a continuing process, which involved themes being linked, grouped, moved, re-labelled, added and removed to produce a set of themes and subthemes and a coding manual, which adequately fitted and thoroughly explained the data. The coding was iteratively developed across lead authors (led by LM and GL) and adjustments made where appropriate based on team discussion. Data saturation was achieved.

Findings

Participants:

For peer review only - http://bmjopen.bmj.com/site/about/guidelines.xhtml

In total 32 participants took part in the study. The age of participants ranged from 18 to 74 (mean age 55). Approximately 72% (23) were women and 28% (9) men.

Themes:

Thematic analysis identified a total of six themes relating to using self-help treatments (steam inhalation/nasal irrigation/other remedies) or consulting with a GP for the treatment of sinusitis. Illustrative quotations are provided and details relating to the interviewee are provided in parentheses.

Table 2: Themes identified in analysis

Themes	Sub-categories
**1. Perceptions of severity**	*-Duration of symptoms*<br>*-Perceived signs of severity*<br>*-Quality of life impact*
**2. Advice from others**	*-Acceptance of GP advice*<br>*-Consideration of alternative advice*
**3. Perceptions of antibiotics**	*-Antibiotics have unpleasant side-effects*<br>*-Concerns about resistance*<br>*-Previous experience of antibiotics.*
**4. Perceptions of self-help treatment**	*-Previous experience of treatments*<br>*-Treatment duration short and irregular*
**5. Experiences of steam inhalation**	*-Beneficial but only for short term relief*<br>*-Beneficial but only for severe symptoms*
**6. Experiences of nasal irrigation**	*-Strong benefits of treatment outweigh any discomfort*<br>*-Uncomfortable side-effects of treatment cannot justify use*

1. Perceptions of severity

Perceptions of severity were shaped by the duration of symptoms, perceived indicators of severity, and particularly by the impact of the illness on quality of life.

Duration of symptoms

For peer review only - http://bmjopen.bmj.com/site/about/guidelines.xhtml

Most participants reported that an evaluation of severity was based on the number of days that they had experienced symptoms, which they related to the current attack (this ranged from a few days to weeks).

"It was horrible, I tried to cope for about a week on my own but in the end I just had to go (to the GP)" (Participant L05).

Perceived signs of severity

A number of factors were used as indicators of severity including a variety of signs and symptoms ranging from pain (head, sinuses, face) to pressure (e.g. nasal passages being blocked), noises (from nose) and sensations (around face and head).

"It's just the….like my face hurting, my headaches and just the…..lethargic and you know just everything about it really" (Participant L04).

Quality of life impact

Participants discussed the way in which their symptoms had prevented them from attending work, caring for their children, and taking part in social or other activities.

"Well, I don't tend to go swimming or anything like that, or a lot of exercise. And even gardening, things where I have to put my head forward, you know, my brain feels like it's bouncing inside my head. So I don't tend to do that sort of thing" (Participant B03).

Others described the sinusitis as having an impact on life in general.

"Well, it literally stops me from having a life when it's really bad because I really can't get up and walk about. So it does interfere with my life" (Participant B10).

2. Advice from others

Acceptance of GP advice

For peer review only - http://bmjopen.bmj.com/site/about/guidelines.xhtml

Overwhelmingly, most participants reported that they were happy to accept and follow their GP's treatment advice. This advice could include taking antibiotics or using various self-help treatments such as nasal irrigation or steam inhalation.

"If I was advised to [use steam inhalation], yes. I mean, it's probably not something I would think about just getting on and doing, but if I was asked- if a GP suggested it then, yes I would" (Participant L06).

Consideration of alternative advice

Sources other than their GP were commonly used in decision making, predominantly family members, but also the internet and newspapers.

"We had relatives over from Australia who brought me a bottle (of eucalyptus oil to inhale) and that's fantastic, that's for a general cold as well" (Participant L05).

3. Perceptions of antibiotics

Individuals' perceptions of antibiotics were related to beliefs about side effects, concerns about resistance, and their previous experience (good or bad) of taking antibiotics.

Antibiotics have unpleasant side-effects

Many participants were well aware of side effects, mainly related to stomach complaints, although could include a variety of effects such as skin rashes, or mouth ulcers.

"*It can give you an upset stomach, well I've experienced it myself, because doesn't it destroy the good bacteria or something?" (Participant B08).*

"Well, ……….they (antibiotics) upset my stomach sometimes." (Participant B10).

"I think some people I've met can't take them, they come out in quite a severe rash sometimes, and I have noticed, perhaps like tiny ulcers appearing in my mouth, which I've found does happen with antibiotics sometimes" (Participant L02)

Concerns about resistance

For peer review only - http://bmjopen.bmj.com/site/about/guidelines.xhtml

Many participants were also aware of, and worried about, the issue of antibiotic resistance, but commonly described it as the body becoming resistant.

> *"We're building up a resistance to them and everything. So I do know there is a problem using them. I felt quite worried when I had to take 2, 100 milligrams in a week" (Participant B10).*

> *"I know it's not the answer because we're all getting immune to them... I am very aware that we can get, well, you know, resistant to antibiotics" (Participant B05).*

Previous experience of antibiotics

Patients who attributed symptom resolution specifically to antibiotics believed they would be effective and might want to take them again for the same symptoms.

> *"I've had a course (of antibiotics) from the doctor and they've been ineffective, and gone back and given me a stronger dose and it does help" (Participant L03).*

> *"Well, only the antibiotics, was the only thing that got rid of it completely" (Participant B04).*

> *"Very often the only thing I can have is an antibiotic to cure it" (Participant B05).*

4. Perceptions of self-help treatment

Previous experience of treatments

Previous experience of using self-help treatments also strongly influenced their decision of whether or not to use them again, to consult a GP, or to try other self-help methods.

> *"Done it (steam inhalation) all my life over the years, so no, no, not a problem doing things like that. It's the way- to be buried under a towel…" (Participant L07).*

> *"It's (inhalation) something I've done for quite a few years with them" (Participant B03).*

For peer review only - http://bmjopen.bmj.com/site/about/guidelines.xhtml

Treatment duration short and irregular

Some participants reported that in general they used self-help treatments only for short and limited periods of time, judging regular and consistent use as unnecessary. Self-help treatments appeared to be used in an irregular and inconsistent way (e.g. stopping treatment (such as steam inhalation) as soon as relief is first experienced).

> *Well I've never used it (steam inhalation) more than probably half a dozen times for one session (illness) over a period of days" (Participant L09)*

5. Experiences of steam inhalation

Beneficial but only for short-term relief

Most participants who had experienced steam inhalation - either as part of the trial, following previous advice from a GP, or as an independent technique which the participant had previously tried - reported that the treatment could be beneficial in relieving symptoms. However, accounts signalled a belief that symptoms were only reduced for a short period of time (up to a few hours).

> *"That steam inhalation does actually provide immediate- it clears the airways, but, again, it just doesn't last. Even if I do it on a regular basis, it's like- you know, within a very short period after doing the inhalation I'm blocked up again…within half an hour" (Participant L05).*

Some participants who had experienced steam inhalation reported that they would only use it if their symptoms were significantly disruptive, positioning steam inhalation as a self-management technique as perhaps better suited for more severe symptoms, with little effect if symptoms were only mild.

> *"I haven't used it (steam inhalation) this time, I suppose it's (sinusitis) not bad enough to use that, but if I couldn't sleep and that, then I would have to do that (inhale steam)" (Participant L04).*

> *"I only do it (steam inhalation) if I'm feeling really bad, if it's getting really bad" (Participant L08)*

For peer review only - http://bmjopen.bmj.com/site/about/guidelines.xhtml

6. Experiences of nasal irrigation

Participants' experiences of using nasal irrigation also influenced their decisions to use the method again or whether to consult a GP. In similar fashion to steam inhalation the treatment of nasal irrigation had been experienced by participants either as part of the trial, but for some following previous advice from a GP, or as an independent technique that the participant had tried previously. Unsurprisingly, it was clear that the balance of discomfort and symptomatic benefit influenced participants' use of irrigation.

Strong benefits of treatment outweigh any discomfort

Approximately half of the participants who had experienced nasal irrigation reported that the technique provided relief for their symptoms and that although the treatment may be slightly uncomfortable at times, the benefits received outweighed any discomfort suffered.

> *"I can put up with it because it does improve the sinus condition" (Participant C01).*

> *"After the initial shock of having to do it, it does help. It helped relieve symptoms…It freaked me out a bit to start with. But, once you get into the hang of it, it's alright" (Participant B09).*

Uncomfortable side-effects of treatment cannot justify use

However, around half of the participants who experienced nasal irrigation reported that the treatment was too uncomfortable or unpleasant. Side-effects reported included; shuddering; an increase in the feeling of mucus being produced, water in the back of the throat, water running out of the nose hours later, and general pain.

> *"One of my memories is of the actual shuddering and in the end I just gave up because I found it very unpleasant to do" (Participant L01).*

> *"It doesn't actually drain it, you can actually blow your nose and get rid of it. It goes everywhere. It goes down the back of your throat and you end up coughing" (Participant B01).*

For peer review only
DRAFT

> *"I don't know whether it's the configuration of the sinuses or whatever, but instead of, sort of, coming out the other side, it was going straight down the tubes into my ears. And, well, that was quite uncomfortable" (Participant B03).*

Discussion

We are aware of no prior qualitative studies of patients' perceptions of steam inhalation and nasal irrigation for recurrent or chronic sinusitis in a primary care setting. The study identified six key themes related to factors that influence patients' decisions to consult a GP or use a self-help treatment (in particular steam inhalation or nasal irrigation) for symptoms of sinusitis. Findings are discussed in relation to the most prevalent and influential themes outlined by participants.

Main findings

Patients with recurrent or chronic sinusitis described the often dramatic impact on their activities and quality of life, and viewed their sinusitis as a chronic condition. They based most treatment decisions on past experiences of managing symptoms, although many were willing to accept GP advice. Thus, although participants were well aware of the limitations of antibiotics and some did not expect to receive them every time they consulted a GP, if they had previously been prescribed antibiotics, they often believed they would be necessary for the future treatment of sinusitis. Patients used self-help treatments for short and limited periods of time only, and did not feel that regular and consistent use was necessary. Interviewees viewed steam inhalation as acceptable and beneficial for symptoms. It was commonly perceived as a method which was only beneficial in providing short term relief, and paradoxically some participants also believed it was only helpful for the most severe symptoms. Similarly, nasal irrigation was generally viewed by interviewees as acceptable and beneficial for symptoms, although some would not use the treatment again due to the balance of uncomfortable or unpleasant side-effects which outweighed any symptom relief. However, within the context of the SNIFS trial detailed information on the correct procedure and potential benefits of both treatments may have helped to increase patient acceptability and adherence in those patients who experienced discomfort.

Comparison with existing literature

For peer review only
DRAFT

If participants had been prescribed antibiotics in the past for the treatment of sinusitis, they attributed symptom benefit to antibiotics and accounts indicated beliefs that this treatment would, therefore, be the optimal approach for future management of the condition. In addition, participants also reported a strong acceptance of any GP advice, also suggesting that if GPs prescribe antibiotics for sinusitis, patients' beliefs that antibiotics may be needed in the future will be strongly reinforced. These findings are supported by previous quantitative research which has documented that prescribing antibiotics for RTIs directly influences patients' views relating to the need to consult a GP and take antibiotics for future RTIs[16].

One factor maintaining high prescription rates for sinusitis (with more than 90% of individuals receiving antibiotics[2]) is likely to be the significant impact of the condition on the patient's quality of life – described in this study in such dramatic terms as 'horrible' and 'it literally stops me from having a life'. The significance of impact on quality of life is supported by similar findings from a secondary care sample[11]. A further factor is that sinusitis is second only to chest infections in a long duration of each attack – on average just short of three weeks[16]. Thus, given an unpleasant and long lasting condition, if GPs have nothing else to recommend antibiotics are likely to be used.

However, patients were aware of the limitations of antibiotics and many would be happy to accept GP advice not to take them, if this was recommended. This lack of expectation for antibiotics has also been reported in previous research, and in particular often contrasts with GP perceptions of high levels of patient pressure to prescribe [e.g. references 15, 17]. Therefore, the findings suggest that if GPs could reduce their prescribing of antibiotics for sinusitis, many patients will be happy to accept this.

Implications of using steam inhalation and nasal irrigation in clinical practice

Overall, patients reported that steam inhalation was an acceptable treatment for the symptoms of sinusitis. However, many patients believed that steam inhalation was only beneficial for the short-term relief of symptoms and this corresponds with findings from the main clinical trial which found limited benefit[12]. Whilst clearly some patients will feel they get benefit from steam inhalation there is also some evidence form other studies of mild thermal injury using steam[18]. Overall, the combined qualitative and quantitative findings suggest that there

For peer review only - http://bmjopen.bmj.com/site/about/guidelines.xhtml

is a limited place for routinely advising patients to use steam inhalation for chronic or recurrent sinusitis.

Patients who had experienced nasal irrigation could be identified in one of two sub-groups: those who described irrigation as an acceptable technique which could relieve their symptoms; and those who described the discomfort experienced during irrigation with limited justification for its use as a treatment. Patients who reportedly found irrigation to be acceptable often reported that they had persisted with it despite initially finding the treatment very uncomfortable, and many reported how they had 'got used to' the discomfort in order to experience the benefits. Therefore, it is possible that the patients who found irrigation unacceptable may have changed their mind if they had persisted with the treatment. In addition, patients who reported irrigation as being unacceptable tended to report side-effects such as water going down their throat. In short, findings suggest that nasal irrigation can be viewed as an acceptable treatment for the symptoms of sinusitis. An important caveat to this, however, is the need for detailed and clear patient information on the correct procedure and the potential benefits of persisting with the technique in terms of finding it easier with increased practice/use of nasal irrigation. Clinicians are recommended to give greater support to patients in using the technique, given the effect shown in the trial (modest benefit at 3 and 6 months from nasal irrigation).

Strengths and limitations

One of the key strengths of the study lies in the fact that all patients interviewed had experienced at least one of the treatment options (steam inhalation or nasal irrigation) as part of the SNIFS trial. Therefore, the interviewees could discuss in-depth the various treatment options in accordance with the way in which the treatments were delivered during the trial.

However, the sample of participants who took part in the interviews may have limited the scope of the findings due to the fact that they had all consented to and taken part in the SNIFS trial. Trial participation may have led to a sample of participants who were particularly interested in research of this nature and may not have represented views held by non-trial patients. In particular, participants may have held more positive views towards self-management treatments due to their interest and participation in research of this nature.

For peer review only - http://bmjopen.bmj.com/site/about/guidelines.xhtml

Despite this possible 'research biased' sample, all patients interviewed did represent a sample of patients who had experienced recurrent sinusitis and had also managed their condition using a number of treatment options over the years. In addition, a few patients interviewed had also withdrawn from the trial but were happy to take part in an interview; suggesting that a wider range of patient views were included within the sample (and not simply those who may view self-management treatments in a more positive light).

Conclusion

The findings from this qualitative study suggest that steam inhalation is viewed as an acceptable treatment which patients are happy to use, although many perceive it as having limited short-term benefit. Nasal irrigation is acceptable to many patients in relieving symptoms, but some find it uncomfortable or mildly unpleasant. However, detailed information on the correct procedure and potential benefits of persisting with the technique may increase the acceptability of nasal irrigation in patients who find it initially uncomfortable, a finding supported by the main trial data.

Contributorship

GM Leydon led the qualitative work as part of the SNIFS trial. P Little secured research funding/and acted as overall PI of the SNIFS trial. T Thomas facilitated coordination and recruitment with P Alexant and S Johnson. L McDermott, S Petley, B Holdstock-Brown, C Wiseman collected interview data. All authors were involved in/commented on data analysis (led by GM Leydon and L McDermott). AH progressed the manuscript and all authors contributed to its writing.

Funding

The study was funded by a grant from the National Institute for Health Research (NIHR) under its Programme Grants for Applied Research (grant no. RP-PG-0407-10098). The views expressed are those of the authors and not necessarily those of the National Health Service, the NIHR or the Department of Health. The University of Southampton was the sponsor, but it had no role in the running of the study, the analysis or interpretation of the results, or the preparation of the manuscript.

For peer review only - http://bmjopen.bmj.com/site/about/guidelines.xhtml

Ethics

This study was given ethical approval by the Hampshire Rec B Research Ethics Committee (number 07/Q1704/69).

Competing interests statement

There are no competing interests.

Acknowledgements

The authors are grateful to the patients and health care professionals who contributed their time and effort and helpful insights to make the study possible. They are also grateful to the Programme Steering Committee for their support and advice throughout the study; NIHR for funding Geraldine Leydon on a personal Fellowship during this study; and C Smith who contributed to the research whilst a medical student.

Data sharing statement

There are no data sets available for sharing.

References

1. Tan T, Little P, Stokes T: Antibiotic prescribing for self limiting respiratory tract infections in primary care: summary of NICE guidance. *BMJ.* 2008, *337:*a437
2. Gulliford MC, Dregan A, Moore MV, et al.: Continued high rates of antibiotic prescribing to adults with respiratory tract infection: survey of 568 UK general practices. *BMJ Open.* 2014;*4:*e006245.
3. Little P, Del Mar C, Gould C, et al.: Primary carePredictors of poor outcome and benefits from antibiotics in children with acute otitis media: pragmatic randomised trialCommentary: research directions for treatment for acute otitis media. *BMJ.* 2002;*325:*22.
4. Macfarlane J, Holmes W, Gard P, et al.: Prospective study of the incidence, aetiology and outcome of adult lower respiratory tract illness in the community. *Thorax.* 2001;*56:*109-114.
5. Dagan R, Leibovitz E, Greenberg D, et al.: Dynamics of pneumococcal nasopharyngeal colonization during the first days of antibiotic treatment in pediatric patients. *Pediatr Infect Dis J.* 1998;*17:*880-885.

For peer review only - http://bmjopen.bmj.com/site/about/guidelines.xhtml

6. Costelloe C, Metcalfe C, Lovering A, Mant D, Hay AD: Effect of antibiotic prescribing in primary care on antimicrobial resistance in individual patients: systematic review and meta-analysis. *BMJ.* 2010;*340:*c2096.
7. Leung E, Weil DE, Raviglione M, Nakatani H: The WHO policy package to combat antimicrobial resistance. *Bulletin of the World Health Organization.* 2011, *89:*390-392.
8. Kassel JC, King D, Spurling GK: Saline nasal irrigation for acute upper respiratory tract infections. *Cochrane Database Syst* Rev. 2010; 17(3):CD006821
9. Tomooka LT, Murphy C, Davidson TM: Clinical study and literature review of nasal irrigation. *Laryngoscope.* 2000;*110:*1189-1193.
10. Benninger MS, Anon J, Mabry RL: The medical management of rhinosinusitis. *Otolaryngol Head Neck Surg.* 1997, *117:*S41-S49.
11. Erskine S, Verkerk M, Notley C, Williamson I, Philpott C: Chronic rhinosinusitis: patient experiences of primary and secondary care–a qualitative study. *Clinical Otolaryngology.* 2016, *41:*8-14.
12. Little P, Stuart B, Mullee M, et al.: Effectiveness of steam inhalation and nasal irrigation for chronic or recurrent sinus symptoms in primary care: a pragmatic randomized controlled trial. *Can. Med. Assoc. J.* 2016;*188:*940-949.
13. Edwards R, Holland J: *What is qualitative interviewing?*. London: Bloomsbury; 2013.
14. Hammersley M: What's Wrong With Ethnography? London: Routledge, 1992.
15. Braun V, Clarke V: Using thematic analysis in psychology. *Qual Red Psychol* 2006;*3:*77-101.
16. Little P, Rumsby K, Kelly J, et al.: Information leaflet and antibiotic prescribing strategies for acute lower respiratory tract infection: a randomized controlled trial. *JAMA.* 2005, *293:*3029-3035.
17. Thompson M, Vodicka TA, Blair PS, et al.: Duration of symptoms of respiratory tract infections in children: systematic review. *BMJ.* 2013, *347:*f7027.
18. Leydon GM, McDermott L, Moore M, et al.: A qualitative study of GP, NP and patient views about the use of rapid streptococcal antigen detection tests (RADTs) in primary care:'swamped with sore throats?'. *BMJ Open.* 2013, *3:*e002460.

For peer review only - http://bmjopen.bmj.com/site/about/guidelines.xhtml

Appendix 1 – interview guide

SNIFS STUDY
Steam inhalation and Nasal Irrigation For recurrent Sinusitis
REC 07/Q1704/69

Part one: Experiences of Sinusitis

1. **Could you tell me how severe you feel your sinus problem is to you?**

   Prompts:
   A: What particularly bothers you about your sinus problems?
   B: Can you explain to me why this affects you?

2. **Could you tell me how often you become affected by your sinus problem?**

   Prompts:
   A: Are they a problem everyday?
   B: When did they first become a problem?
   C: Do you have times when your sinus symptoms don't affect you?
   D: Does your sinus problem prevent you from doing anything? (details of this/how does this make you feel).

3. **Could you tell me how you feel about visiting the doctors about your sinus problems?**

   Prompts:
   A: Have you seen any other healthcare professional about your symptoms? (nurse etc)
   B: Do you find it helpful to visit a doctor about your symptoms?

4. **Do you have any ideas about what could be causing your particular sinus problems?**

   Prompts
   A: Do you think it is something to do with how you are made?
   B: Do you think it is to do with bacteria and viruses?

For peer review only - http://bmjopen.bmj.com/site/about/guidelines.xhtml

Part two: Experiences and views of treatments

6. What treatments have you used to treat your sinus problems in the past?

Prompts:
A: Can you think of something that worked well?
B: Can you think of something that didn't help at all?
C: Where did you get the information from for these treatments?

7. Before this study, had you ever heard about using nasal irrigation?

Prompts
A: How do you feel about it?
B: Do you think it would help to relieve symptoms?
C: Do you think it might have any side effects?
D: How often do you think it should be used for?
E: How have you found using nasal irrigation?

8. Had you heard about using steam inhalation to treat sinusitis before taking part in the study?

Prompts
A: How do you feel about it?
B: Do you think it would help to relieve symptoms?
C: Do you think it might have any side effects?
D: How often do you think it should be used for?
E. How have you found using steam?

9. Have you ever had antibiotics for your sinus problems?

Prompts
A: How did you find using antibiotics?
B: Would you consider using antibiotics again for your sinus problems?
C: Have you heard of any problems with using antibiotics, such as side effects.
D: Have you heard about bacteria becoming resistant to antibiotics?
E: Where did you hear about these issues (tv, newspapers, GP, others etc)

Conclusion

10. Are there any other relevant issues we haven't covered that you would like to mention?

11. Are there any questions you that would like to ask me?

DRAFT For peer review only

COREQ (COnsolidated criteria for REporting Qualitative research) Checklist

A checklist of items that should be included in reports of qualitative research. You must report the page number in your manuscript where you consider each of the items listed in this checklist. If you have not included this information, either revise your manuscript accordingly before submitting or note N/A.

Topic	Item No.	Guide Questions/Description	Reported on Page No.
**Domain 1: Research team and reflexivity**			
Personal characteristics			
Interviewer/facilitator	1	Which author/s conducted the interview or focus group?	4
Credentials	2	What were the researcher's credentials? E.g. PhD, MD	5
Occupation	3	What was their occupation at the time of the study?	5
Gender	4	Was the researcher male or female?	5
Experience and training	5	What experience or training did the researcher have?	5
Relationship with participants			
Relationship established	6	Was a relationship established prior to study commencement?	N/A
Participant knowledge of the interviewer	7	What did the participants know about the researcher? e.g. personal goals, reasons for doing the research	N/A
Interviewer characteristics	8	What characteristics were reported about the inter viewer/facilitator? e.g. Bias, assumptions, reasons and interests in the research topic	N/A
**Domain 2: Study design**			
Theoretical framework			
Methodological orientation and Theory	9	What methodological orientation was stated to underpin the study? e.g. grounded theory, discourse analysis, ethnography, phenomenology, content analysis	5
Participant selection			
Sampling	10	How were participants selected? e.g. purposive, convenience, consecutive, snowball	4
Method of approach	11	How were participants approached? e.g. face-to-face, telephone, mail, email	4
Sample size	12	How many participants were in the study?	5
Non-participation	13	How many people refused to participate or dropped out? Reasons?	N/A
Setting			
Setting of data collection	14	Where was the data collected? e.g. home, clinic, workplace	4
Presence of non-participants	15	Was anyone else present besides the participants and researchers?	N/A
Description of sample	16	What are the important characteristics of the sample? e.g. demographic data, date	Not available
Data collection			
Interview guide	17	Were questions, prompts, guides provided by the authors? Was it pilot tested?	19
Repeat interviews	18	Were repeat inter views carried out? If yes, how many?	N/A
Audio/visual recording	19	Did the research use audio or visual recording to collect the data?	4
Field notes	20	Were field notes made during and/or after the inter view or focus group?	N/A
Duration	21	What was the duration of the inter views or focus group?	4
Data saturation	22	Was data saturation discussed?	5
Transcripts returned	23	Were transcripts returned to participants for comment and/or	N/A

For peer review only

For peer review only - http://bmjopen.bmj.com/site/about/guidelines.xhtml

Topic	Item No.	Guide Questions/Description	Reported on Page No.
		correction?	
**Domain 3: analysis and findings**			
Data analysis			
Number of data coders	24	How many data coders coded the data?	4
Description of the coding tree	25	Did authors provide a description of the coding tree?	No
Derivation of themes	26	Were themes identified in advance or derived from the data?	5
Software	27	What software, if applicable, was used to manage the data?	N/A
Participant checking	28	Did participants provide feedback on the findings?	No
Reporting			
Quotations presented	29	Were participant quotations presented to illustrate the themes/findings? Was each quotation identified? e.g. participant number	6 onwards
Data and findings consistent	30	Was there consistency between the data presented and the findings?	6 onwards
Clarity of major themes	31	Were major themes clearly presented in the findings?	6 onwards
Clarity of minor themes	32	Is there a description of diverse cases or discussion of minor themes?	6 onwards

Developed from: Tong A, Sainsbury P, Craig J. Consolidated criteria for reporting qualitative research (COREQ): a 32-item checklist for interviews and focus groups. *International Journal for Quality in Health Care*. 2007. Volume 19, Number 6: pp. 349 – 357

Once you have completed this checklist, please save a copy and upload it as part of your submission. DO NOT include this checklist as part of the main manuscript document. It must be uploaded as a separate file.

For peer review only

BMJ Open

'Well, it literally stops me from having a life when it's really bad' : a nested qualitative interview study of patient views on the use of self-management treatments for the management of recurrent sinusitis (SNIFS trial).

Journal:	*BMJ Open*
Manuscript ID	bmjopen-2017-017130.R2
Article Type:	Research
Date Submitted by the Author:	13-Jul-2017
Complete List of Authors:	Leydon, Gerry; University of Southampton, Primary Care and Population Sciences<br>McDermott, Lisa; King's College London, Primary Care and Public Health Sciences<br>Thomas, Tammy; University of Southampton, PCPS Unit<br>Halls, Amy; University of Southampton, Primary Care and Population Sciences<br>Holdstock-Brown, Ben; University of Southampton, Primary Care and Population Sciences<br>Petley, Stephen; University of Southampton, Primary Care and Population Sciences<br>Wiseman, Clare; University of Southampton, Primary Care and Population Sciences<br>Little, Paul; University of Southampton, Primary Care and Population Science;
**Primary Subject Heading**:	General practice / Family practice
Secondary Subject Heading:	Qualitative research
Keywords:	PRIMARY CARE, QUALITATIVE RESEARCH, sinusitis, antibiotics, self help

SCHOLARONE™
Manuscripts

'Well, it literally stops me from having a life when it's really bad': a nested qualitative interview study of patient views on the use of self-management treatments for the management of recurrent sinusitis (SNIFS trial)

GM Leydon*±, L McDermott**±, T Thomas*, A Halls*, B Holdstock-Brown*, S Petley*, C Wiseman*, P Little*, on behalf of the SNIFS Investigators

±These authors to be regarded as Joint First Authors.

*Primary Care and Population Sciences, Faculty of Medicine, University of Southampton Aldermoor Health Centre, Aldermoor Close, Southampton, SO16 5ST

**Primary Care and Public Health Sciences, King's College, London 614, 6th Floor, Capital House, 42 Weston Street, London, SE1 3QD

Corresponding author:
Dr GM Leydon
gerry@soton.ac.uk

SNIFS Investigators:
University of Southampton: Paul Little, Tammy Thomas, Pat Alexant, Sophie Johnson, Lisa McDermott, Geraldine M. Leydon, Stephen Petley, Chris Smith, Clare Wiseman.

For peer review only

Abstract

Objective: To explore the experience and perceptions of illness, the decision to consult a GP and the use of self-management approaches for chronic or recurrent sinusitis.

Design: Qualitative semi-structured interview study.

Setting: UK Primary care.

Participants: 32 participants who had been participating in the 'SNIFS' (Steam-inhalation-and- Nasal-Irrigation-For-recurrent-Sinusitis) trial in the South of England.

Method: Thematic analysis of semi-structured, telephone interviews.

Results: Participants often reported dramatic impact on both activities and their quality of life. Participants were aware of both antibiotic side effects and resistance, but if they had previously been prescribed antibiotics, many patients believed that they would be necessary for the future treatment of sinusitis. Participants used self-help treatments for short and limited periods of time only. In the context of the trial, steam inhalation used for recurrent sinusitis was described as acceptable, but is seen as having limited effectiveness. Nasal irrigation was viewed as acceptable and beneficial by more patients. However, some participants reported that they would not use the treatment again due to the uncomfortable side-effects they experienced, which outweighed any symptom relief which may have resulted had they continued.

Conclusions: Steam inhalation is acceptable but seen as having limited effectiveness. Nasal irrigation is generally acceptable and beneficial for symptoms, but detailed information on the correct procedure and potential benefits of persisting may increase acceptability and adherence in those patients who find it uncomfortable.

Strengths and limitations

1. The interviews permitted an exploration of participants' perceptions of particular management strategies for recurrent or chronic sinusitis.
2. Participants represented a sample of patients with recurrent sinusitis who had managed their condition using different treatment options.
3. Trial participation may have led to a sample of participants particularly interested in this research, and therefore may not be representative of 'typical' patients.

For peer review only

4. Due to study procedure limitations the team was unable to collect characteristics of all participants and this has reduced our ability to analyse data according to key characteristics, such as trial arm, in comparative ways.

Background

Many respiratory tract infections (RTIs) are acute and self-limiting conditions, lasting for around one to two weeks. However antibiotic prescriptions for RTIs account for over 60% of all antibiotic prescriptions in primary care[1]. One study of general practice prescribing found antibiotics to be prescribed in around 54% of all consultations for RTIs[2]. Despite the large number of antibiotics frequently prescribed for RTIs, evidence has consistently demonstrated the limited benefit of antibiotics in treating these conditions[1,3,4]. Furthermore, the overuse of antibiotics can contribute to the spread of resistant bacteria[5,6]. This problem is currently on the increase and has been identified by the World Health Organisation as a serious issue which must be addressed with urgency[7].

Whilst antibiotics may be beneficial for some patients with sinusitis, the NICE guidelines recommend that antibiotics should not be prescribed for RTIs in most instances, unless a patient meets a specific at-risk criteria, such as being systematically unwell, or at high risk of serious complications due to a pre-existing comorbidity[1]. However, a number of 'self-help' treatments can be recommended to assist in relieving symptoms related to RTIs[1]. In relation to sinusitis (which is classified as a RTI), there is some evidence to show that both steam inhalation (involving a patient inhaling steam over a bowl of boiling water) and nasal irrigation (the irrigation of nasal cavities with saline) can reduce symptoms. However, the evidence is inconsistent and limited[8,9,10].

Previous qualitative work among patients with chronic sinusitis in secondary care suggests significant impact on quality of life and dissatisfaction with treatments received[11]. However, most recurrent or chronic sinusitis is managed in primary care, and we are aware of no qualitative studies to explore patients' perceptions in primary care. The SNIFS (Steam inhalation and Nasal Irrigation For recurrent Sinusitis) trial aimed to assess the efficacy of steam inhalation and nasal irrigation for the treatment of sinusitis. The study recruited 871 patients from across 72 practices in the South of England. Participants were randomly assigned to an advice strategy in a 2x2 factorial design: to receive advice to use nasal saline irrigation daily, or no such advice; each of these groups was also randomised to receive

For peer review only - http://bmjopen.bmj.com/site/about/guidelines.xhtml

advice to use steam inhalation daily, or no such advice. This advice is shown in Table 1. All study participants had access to usual care. Participants had had at least two episodes of acute sinusitis or one episode of chronic sinusitis (lasting for 12 weeks or more) in the three years before enrolment[12].

Table 1 - SNIFS trial advice strategies

Advice strategy	Definition
Nasal saline irrigation	Participants given verbal instructions and a link to a demonstration video on YouTube. Participants provided with a neti pot and asked to irrigate their nose daily for six months.
Steam inhalation	Participants were asked to inhale steam for five minutes each day.
Usual care	Use of medications or referral was at discretion of patient's physician.

The main findings from the trial have been published and demonstrate modest benefit at three and six months from nasal irrigation but not for steam inhalation[12]. This paper reports on the findings of a nested qualitative study which aimed to explore the feasibility and acceptability of these treatments from the patient perspective, the experience of illness and previous treatments, and factors which influence patient decisions to consult a GP or use self-help treatments (including steam inhalation and nasal irrigation) for the symptoms of sinusitis.

Methods

Participants and procedure

Participants were recruited from patients with chronic or recurrent sinusitis who were taking part in the SNIFS trial. Patients had seen the GP for previous episodes of sinusitis, and were also experiencing symptoms currently, although they did not have any 'at risk criteria' as this would have excluded them from participating in the trial. Participants in the trial consented to being invited by telephone for an interview. Participants who had withdrawn from the trial were also eligible to take part in the interviews. The participants were recruited from areas across Southampton and Hampshire. Interview participants were purposively sampled to include a range of participants according to their allocation in the trial (e.g. steam inhalation/ nasal irrigation), and age and gender.

For peer review only - http://bmjopen.bmj.com/site/about/guidelines.xhtml

Interviews

Trained interviewers LM and CW (female) and SP and BH (male) conducted telephone interviews (in order to include a wide geographical area), with each lasting approximately half an hour. Ethical approval was in place for interviews to last up to 60 minutes and no interviews exceeded this. SP, BH and CW were medical students during their involvement in this research, and supervised by senior academics (GL and PL). Each participant was interviewed once, and all interviews were audio–recorded and transcribed verbatim in preparation for analysis. Semi-structured qualitative interviews allowed researchers to gather insights into participants' individual views and experiences of treatments for sinusitis, as well as providing a structure for comparison across different accounts[13]. The interview guide (Appendix 1, supplementary file) was developed as part of a student project and tested with a member of staff: this provided a training opportunity for interviewing and feedback on functionality. Previous literature was reviewed and questions thought to be relevant included. It was then refined and agreed by the research team to ensure our aims were met. It included key topic areas while also providing flexibility to explore unanticipated issues. Subtle realism best characterises our epistemological position[14].

Analysis

Inductive thematic analysis[15] was conducted on all transcripts to determine factors that influence patients' decision to consult a GP or use an alternative treatment for sinusitis, as well as being open to themes outside of the core aims of the study. Following immersion in the transcripts, familiarisation was achieved and recurrent patterns and prominent themes were identified and labelled with codes. Each code label referred directly to the operationalisation of the theme content. A label and full descriptive definition was then provided for each theme. The codes and definitions were refined during a continuing process, which involved themes being linked, grouped, moved, re-labelled, added and removed to produce a set of themes and subthemes and a coding manual, which adequately fitted and thoroughly explained the data. The coding was iteratively developed across lead authors (led by LM and GL) and adjustments made where appropriate based on team discussion. Data saturation[16] was achieved and recruitment ceased, with no further interviews conducted.

For peer review only - http://bmjopen.bmj.com/site/about/guidelines.xhtml

Findings

Participants:

In total 32 participants took part in the study. The age of participants ranged from 18 to 74 (mean age 55). Approximately 72% (23) were women and 28% (9) men.

Themes:

Thematic analysis identified a total of six themes relating to using self-help treatments (steam inhalation/nasal irrigation/other remedies) or consulting with a GP for the treatment of sinusitis. Illustrative quotations are provided and details relating to the interviewee are provided in parentheses. The themes are shown in Table 2.

Table 2: Themes identified in analysis

Themes	Sub-categories
**1. Perceptions of severity**	*-Duration of symptoms*<br>*-Perceived signs of severity*<br>*-Quality of life impact*
**2. Advice from others**	*-Acceptance of GP advice*<br>*-Consideration of alternative advice*
**3. Perceptions of antibiotics**	*-Antibiotics have unpleasant side-effects*<br>*-Concerns about resistance*<br>*-Previous experience of antibiotics.*
**4. Perceptions of self-help treatment**	*-Previous experience of treatments*<br>*-Treatment duration short and irregular*
**5. Experiences of steam inhalation**	*-Beneficial but only for short term relief*<br>*-Beneficial but only for severe symptoms*
**6. Experiences of nasal irrigation**	*-Strong benefits of treatment outweigh any discomfort*<br>*-Uncomfortable side-effects of treatment cannot justify use*

1. Perceptions of severity

Perceptions of severity were shaped by the duration of symptoms, perceived indicators of severity, and particularly by the impact of the illness on quality of life.

For peer review only - http://bmjopen.bmj.com/site/about/guidelines.xhtml

Duration of symptoms

Most participants reported that an evaluation of severity was based on the number of days that they had experienced symptoms, which they related to the current attack (this ranged from a few days to weeks).

> *"It was horrible, I tried to cope for about a week on my own but in the end I just had to go (to the GP)" (Participant L05).*

Perceived signs of severity

A number of factors were used as indicators of severity including a variety of signs and symptoms ranging from pain (head, sinuses, face) to pressure (e.g. nasal passages being blocked), noises (from nose) and sensations (around face and head).

> *"It's just the....like my face hurting, my headaches and just the.....lethargic and you know just everything about it really" (Participant L04).*

Quality of life impact

Participants discussed the way in which their symptoms had prevented them from attending work, caring for their children, and taking part in social or other activities.

> *"Well, I don't tend to go swimming or anything like that, or a lot of exercise. And even gardening, things where I have to put my head forward, you know, my brain feels like it's bouncing inside my head. So I don't tend to do that sort of thing" (Participant B03).*

Others described the sinusitis as having an impact on life in general.

> *"Well, it literally stops me from having a life when it's really bad because I really can't get up and walk about. So it does interfere with my life" (Participant B10).*

2. Advice from others

For peer review only

Acceptance of GP advice

Overwhelmingly, most participants reported that they were happy to accept and follow their GP's treatment advice. This advice could include taking antibiotics or using various self-help treatments such as nasal irrigation or steam inhalation.

> *"If I was advised to [use steam inhalation], yes. I mean, it's probably not something I would think about just getting on and doing, but if I was asked- if a GP suggested it then, yes I would" (Participant L06).*

Consideration of alternative advice

Sources other than their GP were commonly used in decision making, predominantly family members, but also the internet and newspapers.

> *"We had relatives over from Australia who brought me a bottle (of eucalyptus oil to inhale) and that's fantastic, that's for a general cold as well" (Participant L05).*

3. Perceptions of antibiotics

Individuals' perceptions of antibiotics were related to beliefs about side effects, concerns about resistance, and their previous experience (good or bad) of taking antibiotics.

Antibiotics have unpleasant side-effects

Many participants were well aware of side effects, mainly related to stomach complaints, although could include a variety of effects such as skin rashes, or mouth ulcers.

> "*It can give you an upset stomach, well I've experienced it myself, because doesn't it destroy the good bacteria or something?" (Participant B08).*

> *"Well, ………....they (antibiotics) upset my stomach sometimes." (Participant B10).*

> *"I think some people I've met can't take them, they come out in quite a severe rash sometimes, and I have noticed, perhaps like tiny ulcers appearing in my mouth, which I've found does happen with antibiotics sometimes" (Participant L02)*

For peer review only - http://bmjopen.bmj.com/site/about/guidelines.xhtml

Concerns about resistance

Many participants were also aware of, and worried about, the issue of antibiotic resistance, but commonly described it as the body becoming resistant.

> *"We're building up a resistance to them and everything. So I do know there is a problem using them. I felt quite worried when I had to take 2, 100 milligrams in a week" (Participant B10).*

> *"I know it's not the answer because we're all getting immune to them... I am very aware that we can get, well, you know, resistant to antibiotics" (Participant B05).*

Previous experience of antibiotics

Patients who attributed symptom resolution specifically to antibiotics believed they would be effective and might want to take them again for the same symptoms.

> *"Well, only the antibiotics, was the only thing that got rid of it completely" (Participant B04).*

> *"Very often the only thing I can have is an antibiotic to cure it" (Participant B05).*

> *"I've got to have antibiotics" (Participant B05)*

> *"Well, I got to the point where I just used to ring them up and say, "I know what I've got – can I have some antibiotics please?"" (Participant B04).*

4. Perceptions of self-help treatment

Previous experience of treatments

Previous experience of using self-help treatments also strongly influenced their decision of whether or not to use them again, to consult a GP, or to try other self-help methods.

For peer review only - http://bmjopen.bmj.com/site/about/guidelines.xhtml

"Done it (steam inhalation) all my life over the years, so no, no, not a problem doing things like that. It's the way- to be buried under a towel…" (Participant L07).

"It's (inhalation) something I've done for quite a few years with them" (Participant B03).

Treatment duration short and irregular

Some participants reported that in general they used self-help treatments only for short and limited periods of time. Self-help treatments appeared to be used in an irregular and inconsistent way (e.g. stopping treatment (such as steam inhalation) as soon as relief is first experienced).

Sometimes I think perhaps I've – that is excessive [using nasal irrigation twice a day], especially now that I feel everything is clearer, but particularly to start with, that was quite a relief' (Participant B06).

Well I've never used it (steam inhalation) more than probably half a dozen times for one session (illness) over a period of days" (Participant L09)

5. Experiences of steam inhalation

Beneficial but only for short-term relief

Most participants who had experienced steam inhalation - either as part of the trial, following previous advice from a GP, or as an independent technique which the participant had previously tried - reported that the treatment could be beneficial in relieving symptoms. However, accounts signalled a belief that symptoms were only reduced for a short period of time (up to a few hours).

"That steam inhalation does actually provide immediate- it clears the airways, but, again, it just doesn't last. Even if I do it on a regular basis, it's like- you know, within a very short period after doing the inhalation I'm blocked up again…within half an hour" (Participant L05).

For peer review only

Some participants who had experienced steam inhalation reported that they would only use it if their symptoms were significantly disruptive, positioning steam inhalation as a self-management technique as perhaps better suited for more severe symptoms..

> *"I haven't used it (steam inhalation) this time, I suppose it's (sinusitis) not bad enough to use that, but if I couldn't sleep and that, then I would have to do that [inhale steam]" (Participant L04).*

> *"I only do it (steam inhalation) if I'm feeling really bad, if it's getting really bad" (Participant L08)*

For peer review only

6. Experiences of nasal irrigation

Participants' experiences of using nasal irrigation also influenced their decisions to use the method again or whether to consult a GP. In similar fashion to steam inhalation the treatment of nasal irrigation had been experienced by participants either as part of the trial, but for some following previous advice from a GP, or as an independent technique that the participant had tried previously. Unsurprisingly, it was clear that the balance of discomfort and symptomatic benefit influenced participants' use of irrigation.

Strong benefits of treatment outweigh any discomfort

Some of the participants who had experienced nasal irrigation reported that the technique provided relief for their symptoms and that although the treatment may be slightly uncomfortable at times, the benefits received outweighed any discomfort suffered.

> *"I can put up with it because it does improve the sinus condition" (Participant C01).*

> *"After the initial shock of having to do it, it does help. It helped relieve symptoms…It freaked me out a bit to start with. But, once you get into the hang of it, it's alright" (Participant B09).*

Uncomfortable side-effects of treatment cannot justify use

However, around half of the participants who experienced nasal irrigation reported that the treatment was too uncomfortable or unpleasant. Side-effects reported included;

shuddering; an increase in the feeling of mucus being produced, water in the back of the throat, water running out of the nose hours later, and general pain.

"One of my memories is of the actual shuddering and in the end I just gave up because I found it very unpleasant to do" (Participant L01).

"It doesn't actually drain it, you can actually blow your nose and get rid of it. It goes everywhere. It goes down the back of your throat and you end up coughing" (Participant B01).

"I don't know whether it's the configuration of the sinuses or whatever, but instead of, sort of, coming out the other side, it was going straight down the tubes into my ears. And, well, that was quite uncomfortable" (Participant B03).

Discussion

We are aware of no prior qualitative studies of patients' perceptions of steam inhalation and nasal irrigation for recurrent or chronic sinusitis in a primary care setting. The study identified six key themes related to factors that influence patients' decisions to consult a GP or use a self-help treatment (in particular steam inhalation or nasal irrigation) for symptoms of sinusitis. Findings are discussed in relation to the most prevalent and influential themes outlined by participants.

Main findings

Patients with recurrent or chronic sinusitis described the often dramatic impact on their activities and quality of life, and viewed their sinusitis as a chronic condition. They based most treatment decisions on past experiences of managing symptoms, although many were willing to accept GP advice. Thus, although many participants were well aware of the potential negative consequences of antibiotics and some did not expect to receive them every time they consulted a GP, if they had previously been prescribed antibiotics, they often believed they would be necessary for the future treatment of sinusitis. Patients generally used self-help treatments for short and limited periods of time only. Interviewees viewed steam inhalation as acceptable and beneficial for symptoms. It was commonly perceived as a method which was only beneficial in providing short term relief, and some participants

For peer review only

believed it was mostly helpful for severe symptoms. Similarly, nasal irrigation was generally viewed by interviewees as acceptable and beneficial for symptoms, although some would not use the treatment again due to the balance of uncomfortable or unpleasant side-effects which outweighed any potential symptom relief. However, within the context of the SNIFS trial detailed information on the correct procedure and potential benefits of both treatments may have helped to increase patient acceptability and adherence in those patients who experienced discomfort.

Comparison with existing literature

If participants had been prescribed antibiotics in the past for the treatment of sinusitis, they attributed symptom benefit to antibiotics and accounts indicated beliefs that this treatment would, therefore, be the optimal approach for future management of the condition. In addition, participants also reported an acceptance of any GP advice, suggesting that if GPs prescribe antibiotics for sinusitis, patients' beliefs that antibiotics may be needed in the future will be strongly reinforced. These findings are supported by previous quantitative research which has documented that prescribing antibiotics for RTIs directly influences patients' views relating to the need to consult a GP and take antibiotics for future RTIs[17].

One factor maintaining high prescription rates for sinusitis (with more than 90% of individuals receiving antibiotics[2]) is likely to be the significant impact of the condition on the patient's quality of life – described in this study in such dramatic terms as 'horrible' and 'it literally stops me from having a life'. The significance of impact on quality of life is supported by similar findings from a secondary care sample[11]. A further factor is that sinusitis is second only to chest infections in a long duration of each attack – on average just short of three weeks[17]. Thus, given an unpleasant and long lasting condition, if GPs have nothing else to recommend antibiotics are likely to be used.

However, patients were aware of the consequences of antibiotics and many would be happy to accept GP advice not to take them, if this was recommended. This lack of expectation for antibiotics has also been reported in research with patients with LRTI and sore throat, and in particular often contrasts with GP perceptions of high levels of patient pressure to prescribe [e.g. references 15, 18]. Therefore, the findings suggest that if GPs could reduce their prescribing of antibiotics for sinusitis, many patients will be happy to accept this.

For peer review only - http://bmjopen.bmj.com/site/about/guidelines.xhtml

Implications of using steam inhalation and nasal irrigation in clinical practice

Overall, patients reported that steam inhalation was an acceptable treatment for the symptoms of sinusitis. However, many patients believed that steam inhalation was only beneficial for the short-term relief of symptoms and this corresponds with findings from the main clinical trial which found limited benefit[12]. Whilst clearly some patients will feel they get benefit from steam inhalation there is also some evidence form other studies of mild thermal injury using steam[19]. Overall, the combined qualitative and quantitative findings suggest that there is a limited place for routinely advising patients to use steam inhalation for chronic or recurrent sinusitis.

Patients who had experienced nasal irrigation could be identified in one of two sub-groups: those who described irrigation as an acceptable technique which could relieve their symptoms; and those who described the discomfort experienced during irrigation with limited justification for its use as a treatment. Patients who reportedly found irrigation to be acceptable often reported that they had persisted with it despite initially finding the treatment very uncomfortable, and many reported how they had 'got used to' the discomfort in order to experience the benefits. Therefore, it is possible that the patients who found irrigation unacceptable may have changed their mind if they had persisted with the treatment. In addition, patients who reported irrigation as being unacceptable tended to report side-effects such as water going down their throat. In short, findings suggest that nasal irrigation can be viewed as an acceptable treatment for the symptoms of sinusitis. An important caveat to this, however, is the need for detailed and clear patient information on the correct procedure and the potential benefits of persisting with the technique in terms of finding it easier with increased practice/use of nasal irrigation. Clinicians are recommended to give greater support to patients in using the technique, given the effect shown in the trial (modest benefit at 3 and 6 months from nasal irrigation).

Strengths and limitations

One of the key strengths of the study lies in the fact that all patients interviewed had experienced at least one of the treatment options (steam inhalation or nasal irrigation) as part of the SNIFS trial. Therefore, the interviewees could discuss in-depth the various treatment options in accordance with the way in which the treatments were delivered during the trial.

For peer review only

However, the sample of participants who took part in the interviews may have limited the scope of the findings due to the fact that they had all consented to and taken part in the SNIFS trial. Trial participation may have led to a sample of participants who were particularly interested in research of this nature and may not have represented views held by non-trial patients. In particular, participants may have held more positive views towards self-management treatments due to their interest and participation in research of this nature.

Despite this possible ‘research biased’ sample, all patients interviewed did represent a sample of patients who had experienced recurrent sinusitis and had also managed their condition using a number of treatment options over the years. In addition, a few patients interviewed had also withdrawn from the trial but were happy to take part in an interview; suggesting that a wider range of patient views were included within the sample (and not simply those who may view self-management treatments in a more positive light).

Conclusion

The findings from this qualitative study suggest that steam inhalation is viewed as an acceptable treatment which patients are happy to use, although many perceive it as having limited short-term benefit. Nasal irrigation is acceptable to many patients in relieving symptoms, but some find it uncomfortable or mildly unpleasant. However, detailed information on the correct procedure and potential benefits of persisting with the technique may increase the acceptability of nasal irrigation in patients who find it initially uncomfortable, a finding supported by the main trial data.

Contributorship

GM Leydon led the qualitative work as part of the SNIFS trial. P Little secured research funding/and acted as overall PI of the SNIFS trial. T Thomas facilitated coordination and recruitment with P Alexant and S Johnson. L McDermott, S Petley, B Holdstock-Brown, C Wiseman collected interview data. All authors were involved in/commented on data analysis (led by GM Leydon and L McDermott). AH and GM Leydon progressed the manuscript and all authors contributed to its writing.

Funding

For peer review only

The study was funded by a grant from the National Institute for Health Research (NIHR) under its Programme Grants for Applied Research (grant no. RP-PG-0407-10098). The views expressed are those of the authors and not necessarily those of the National Health Service, the NIHR or the Department of Health. The University of Southampton was the sponsor, but it had no role in the running of the study, the analysis or interpretation of the results, or the preparation of the manuscript.

For peer review only

Ethics

This study was given ethical approval by the Hampshire Rec B Research Ethics Committee (number 07/Q1704/69).

Competing interests statement

There are no competing interests.

Acknowledgements

The authors are grateful to the patients and health care professionals who contributed their time and effort and helpful insights to make the study possible. They are also grateful to the Programme Steering Committee for their support and advice throughout the study; NIHR for funding Geraldine Leydon on a personal Fellowship during this study; and C Smith who contributed to the research whilst a medical student.

Data sharing statement

There are no data sets available for sharing.

References

1. Tan T, Little P, Stokes T: Antibiotic prescribing for self limiting respiratory tract infections in primary care: summary of NICE guidance. *BMJ.* 2008, *337:*a437
2. Gulliford MC, Dregan A, Moore MV, et al.: Continued high rates of antibiotic prescribing to adults with respiratory tract infection: survey of 568 UK general practices. *BMJ Open.* 2014;*4:*e006245.

3. Little P, Del Mar C, Gould C, et al.: Primary carePredictors of poor outcome and benefits from antibiotics in children with acute otitis media: pragmatic randomised trialCommentary: research directions for treatment for acute otitis media. *BMJ.* 2002;*325:*22.
4. Macfarlane J, Holmes W, Gard P, et al.: Prospective study of the incidence, aetiology and outcome of adult lower respiratory tract illness in the community. *Thorax.* 2001;*56:*109-114.
5. Dagan R, Leibovitz E, Greenberg D, et al.: Dynamics of pneumococcal nasopharyngeal colonization during the first days of antibiotic treatment in pediatric patients. *Pediatr Infect Dis J.* 1998;*17:*880-885.
6. Costelloe C, Metcalfe C, Lovering A, Mant D, Hay AD: Effect of antibiotic prescribing in primary care on antimicrobial resistance in individual patients: systematic review and meta-analysis. *BMJ.* 2010;*340:*c2096.
7. Leung E, Weil DE, Raviglione M, Nakatani H: The WHO policy package to combat antimicrobial resistance. *Bulletin of the World Health Organization.* 2011, *89:*390-392.
8. Kassel JC, King D, Spurling GK: Saline nasal irrigation for acute upper respiratory tract infections. *Cochrane Database Syst* Rev. 2010; 17(3):CD006821
9. Tomooka LT, Murphy C, Davidson TM: Clinical study and literature review of nasal irrigation. *Laryngoscope.* 2000;*110:*1189-1193.
10. Benninger MS, Anon J, Mabry RL: The medical management of rhinosinusitis. *Otolaryngol Head Neck Surg.* 1997, *117:*S41-S49.
11. Erskine S, Verkerk M, Notley C, Williamson I, Philpott C: Chronic rhinosinusitis: patient experiences of primary and secondary care–a qualitative study. *Clinical Otolaryngology.* 2016, *41:*8-14.
12. Little P, Stuart B, Mullee M, et al.: Effectiveness of steam inhalation and nasal irrigation for chronic or recurrent sinus symptoms in primary care: a pragmatic randomized controlled trial. *Can. Med. Assoc. J.* 2016;*188:*940-949.
13. Edwards R, Holland J: *What is qualitative interviewing?*. London: Bloomsbury; 2013.
14. Hammersley M: What's Wrong With Ethnography? London: Routledge, 1992.
15. Braun V, Clarke V: Using thematic analysis in psychology. *Qual Res Psychol* 2006;*3:*77-101.
16. Morse JM: The significance of saturation. *Qualitative Health Research*. 1995;5:147-9.
17. Little P, Rumsby K, Kelly J, et al.: Information leaflet and antibiotic prescribing strategies for acute lower respiratory tract infection: a randomized controlled trial. *JAMA.* 2005, *293:*3029-3035.
18. Thompson M, Vodicka TA, Blair PS, et al.: Duration of symptoms of respiratory tract infections in children: systematic review. *BMJ.* 2013, *347:*f7027.
19. Leydon GM, McDermott L, Moore M, et al.: A qualitative study of GP, NP and patient views about the use of rapid streptococcal antigen detection tests (RADTs) in primary care:'swamped with sore throats?'. *BMJ Open.* 2013, *3:*e002460.

For peer review only

Appendix 1 – interview guide

SNIFS STUDY
Steam inhalation and Nasal Irrigation For recurrent Sinusitis
REC 07/Q1704/69

Part one: Experiences of Sinusitis

1. **Could you tell me how severe you feel your sinus problem is to you?**

   Prompts:
   A: What particularly bothers you about your sinus problems?
   B: Can you explain to me why this affects you?

2. **Could you tell me how often you become affected by your sinus problem?**

   Prompts:
   A: Are they a problem everyday?
   B: When did they first become a problem?
   C: Do you have times when your sinus symptoms don't affect you?
   D: Does your sinus problem prevent you from doing anything? (details of this/how does this make you feel).

3. Could you tell me how you feel about visiting the doctors about your sinus problems?

Prompts:
A: Have you seen any other healthcare professional about your symptoms? (nurse etc)
B: Do you find it helpful to visit a doctor about your symptoms?

4. Do you have any ideas about what could be causing your particular sinus problems?

Prompts
A: Do you think it is something to do with how you are made?
B: Do you think it is to do with bacteria and viruses?

For peer review only

Part two: Experiences and views of treatments

6. What treatments have you used to treat your sinus problems in the past?

Prompts:
A: Can you think of something that worked well?
B: Can you think of something that didn't help at all?
C: Where did you get the information from for these treatments?

7. Before this study, had you ever heard about using nasal irrigation?

Prompts
A: How do you feel about it?
B: Do you think it would help to relieve symptoms?
C: Do you think it might have any side effects?
D: How often do you think it should be used for?
E: How have you found using nasal irrigation?

8. Had you heard about using steam inhalation to treat sinusitis before taking part in the study?

Prompts
A: How do you feel about it?
B: Do you think it would help to relieve symptoms?
C: Do you think it might have any side effects?
D: How often do you think it should be used for?
E. How have you found using steam?

9. Have you ever had antibiotics for your sinus problems?

Prompts
A: How did you find using antibiotics?
B: Would you consider using antibiotics again for your sinus problems?
C: Have you heard of any problems with using antibiotics, such as side effects.
D: Have you heard about bacteria becoming resistant to antibiotics?
E: Where did you hear about these issues (tv, newspapers, GP, others etc)

Conclusion

10. Are there any other relevant issues we haven't covered that you would like to mention?

11. Are there any questions you that would like to ask me?

For peer review only

COREQ (COnsolidated criteria for REporting Qualitative research) Checklist

A checklist of items that should be included in reports of qualitative research. You must report the page number in your manuscript where you consider each of the items listed in this checklist. If you have not included this information, either revise your manuscript accordingly before submitting or note N/A.

Topic	Item No.	Guide Questions/Description	Reported on Page No.
**Domain 1: Research team and reflexivity**			
Personal characteristics			
Interviewer/facilitator	1	Which author/s conducted the interview or focus group?	4
Credentials	2	What were the researcher's credentials? E.g. PhD, MD	5
Occupation	3	What was their occupation at the time of the study?	5
Gender	4	Was the researcher male or female?	5
Experience and training	5	What experience or training did the researcher have?	5
Relationship with participants			
Relationship established	6	Was a relationship established prior to study commencement?	No
Participant knowledge of the interviewer	7	What did the participants know about the researcher? e.g. personal goals, reasons for doing the research	No
Interviewer characteristics	8	What characteristics were reported about the inter viewer/facilitator? e.g. Bias, assumptions, reasons and interests in the research topic	No
**Domain 2: Study design**			
Theoretical framework			
Methodological orientation and Theory	9	What methodological orientation was stated to underpin the study? e.g. grounded theory, discourse analysis, ethnography, phenomenology, content analysis	5
Participant selection			
Sampling	10	How were participants selected? e.g. purposive, convenience, consecutive, snowball	4
Method of approach	11	How were participants approached? e.g. face-to-face, telephone, mail, email	4
Sample size	12	How many participants were in the study?	5
Non-participation	13	How many people refused to participate or dropped out? Reasons?	No
Setting			
Setting of data collection	14	Where was the data collected? e.g. home, clinic, workplace	4
Presence of non-participants	15	Was anyone else present besides the participants and researchers?	4
Description of sample	16	What are the important characteristics of the sample? e.g. demographic data, date	Not available
Data collection			
Interview guide	17	Were questions, prompts, guides provided by the authors? Was it pilot tested?	19
Repeat interviews	18	Were repeat inter views carried out? If yes, how many?	5
Audio/visual recording	19	Did the research use audio or visual recording to collect the data?	4
Field notes	20	Were field notes made during and/or after the inter view or focus group?	No
Duration	21	What was the duration of the inter views or focus group?	4
Data saturation	22	Was data saturation discussed?	5
Transcripts returned	23	Were transcripts returned to participants for comment and/or	No

For peer review only

Topic	Item No.	Guide Questions/Description	Reported on Page No.
		correction?	
**Domain 3: analysis and findings**			
Data analysis			
Number of data coders	24	How many data coders coded the data?	4
Description of the coding tree	25	Did authors provide a description of the coding tree?	No
Derivation of themes	26	Were themes identified in advance or derived from the data?	5
Software	27	What software, if applicable, was used to manage the data?	No
Participant checking	28	Did participants provide feedback on the findings?	No
Reporting			
Quotations presented	29	Were participant quotations presented to illustrate the themes/findings? Was each quotation identified? e.g. participant number	6 onwards
Data and findings consistent	30	Was there consistency between the data presented and the findings?	6 onwards
Clarity of major themes	31	Were major themes clearly presented in the findings?	6 onwards
Clarity of minor themes	32	Is there a description of diverse cases or discussion of minor themes?	6 onwards

Developed from: Tong A, Sainsbury P, Craig J. Consolidated criteria for reporting qualitative research (COREQ): a 32-item checklist for interviews and focus groups. *International Journal for Quality in Health Care*. 2007. Volume 19, Number 6: pp. 349 – 357

Once you have completed this checklist, please save a copy and upload it as part of your submission. DO NOT include this checklist as part of the main manuscript document. It must be uploaded as a separate file.

For peer review only
